# The Suprapyramidal and Infrapyramidal Blades of the Dentate Gyrus Exhibit Different GluN Subunit Content and Dissimilar Frequency‐Dependent Synaptic Plasticity In Vivo

**DOI:** 10.1002/hipo.70002

**Published:** 2025-02-24

**Authors:** Christina Strauch, Juliane Böge, Olena Shchyglo, Valentyna Dubovyk, Denise Manahan‐Vaughan

**Affiliations:** ^1^ Department of Neurophysiology, Medical Faculty Ruhr University Bochum Bochum Germany

**Keywords:** hippocampus, infrapyramidal, NMDA receptor, patch clamp, suprapyramidal

## Abstract

The entorhinal cortex sends afferent information to the hippocampus by means of the perforant path (PP). The PP input to the dentate gyrus (DG) terminates in the suprapyramidal (sDG) and infrapyramidal (iDG) blades. Different electrophysiological stimulation patterns of the PP can generate hippocampal synaptic plasticity. Whether frequency‐dependent synaptic plasticity differs in the sDG and iDG is unclear. Here, we compared medial PP–DG responses in freely behaving adult rats and found that synaptic plasticity in the sDG is broadly frequency dependent, whereby long‐term depression (LTD, > 24 h) is induced with stimulation at 1 Hz, short‐term depression (< 2 h) is triggered by 5 or 10 Hz, and long‐term potentiation (LTP) of increasing magnitudes is induced by 200 and 400 Hz stimulation, respectively. By contrast, although the iDG expresses STD following 5 or 10 Hz stimulation, LTD induced by 1 Hz is weaker, LTP is not induced by 200 Hz and LTP induced by 400 Hz stimulation is significantly smaller in magnitude than LTP induced in sDG. Furthermore, the stimulus–response relationship of iDG is suppressed compared to sDG. These differences may arise from differences in granule cell properties, or the complement of NMDA receptors. Patch clamp recordings, in vitro, revealed reduced firing frequencies in response to high currents, and different action potential thresholds in iDG compared to sDG. Assessment of the expression of GluN subunits revealed significantly lower expression levels of GluN1, GluN2A, and GluN2B in the middle molecular layer of iDG compared to sDG. Taken together, these data indicate that synaptic plasticity in the iDG is weaker, less persistent and less responsive to afferent frequencies than synaptic plasticity in sDG. Effects may be mediated by weaker NMDA receptor expression and differences in neuronal responses in iDG versus sDG. These characteristics may explain reported differences in experience‐dependent information processing in the suprapyramidal and infrapyramidal blades of the DG.

## 1 Introduction

**FIGURE 1:**
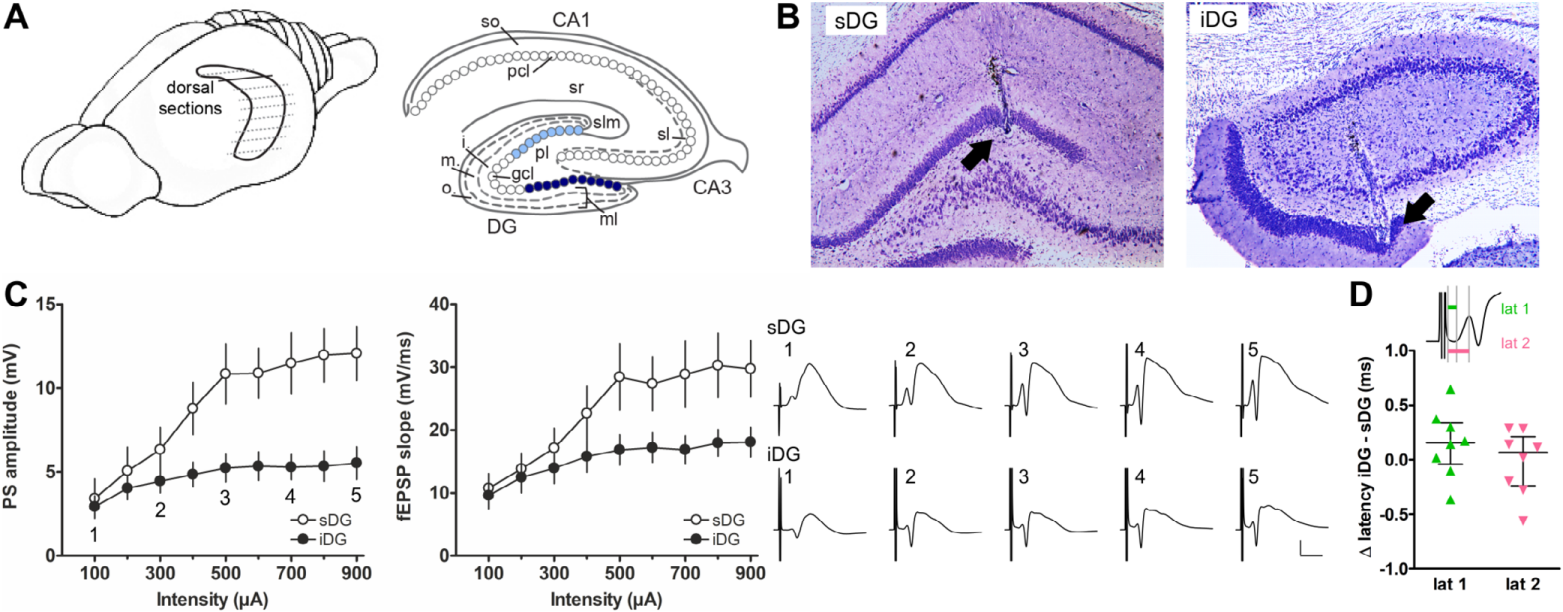
Electrode position and stimulus response relationship of the supra‐ and infrapyramidal blades of the dorsal dentate gyrus. (A) Left: Hippocampal formation depicted within a scheme of the rat brain. For immunohistochemistry, the brain was sectioned in 30 μm thick horizontal sections (dashed lines) and sections of the dorsal hippocampus (continuous line) were used for analysis. Right: Scheme of the hippocampal formation showing the dentate gyrus (DG) and the cornu ammonis subregions CA1 and CA3 as well as the layers of each subregion. NMDAR subunits were analyzed for the granule cell layer (gcl), outer (o.), middle (m.), and inner (i.) molecular layer (ml) of the supra‐ (sDG), and infrapyramidal (iDG) blade of the DG. The sDG (light blue) and iDG (dark blue) of the granule cell layer are highlighted. (B) Left: Nissl‐stained coronal section showing the final position of the recording electrode in the granule cell layer of the sDG (ca. 3.1 mm posterior to bregma according to Paxinos and Watson (2014)). Right: Example of Nissl‐stained coronal section with the final position of the recording electrode in the iDG (ca. 1.9 mm posterior to bregma). The deepest position of each electrode is indicated by an arrow. (C) Evaluation of the stimulus response relationship showing PS and fEPSP of simultaneously evoked responses in sDG and iDG (*N* = 8, each), elicited by test‐pulse stimulation of the medial perforant path. Left: PS of evoked responses are significantly different. Middle: FEPSPs are not significantly different between sDG and iDG. Right: Representative analog examples evoked in each blade obtained at increasing stimulation intensities: (1) 100, (2) 300, (3) 500, (4) 700, and (5) 900 μA. Calibration: Vertical bar: 5 mV, horizontal bar: 5 ms. (D) Evaluation of latencies of evoked responses of the stimulus–response relationship revealed no difference for both the latency to the start of the fEPSP and the latency to the start of the PS. Visualized is the difference for each latency in iDG and sDG of each animal. Inset depicts how latencies were calculated on an enlarged excerpt of an analog example.

The regions of the hippocampal formation, comprising the cornus ammonis (CA) and dentate gyrus (DG), can be functionally differentiated along the dorsoventral axis (Fanselow and Dong 2010; Moser and Moser 1998). Within these compartments they can be further subdivided (Chawla et al. 2005; Henriksen et al. 2010; Hoang, Aliane, and Manahan‐Vaughan 2018; Witter et al. 2000). For example, the DG can be segregated into at least two anatomical subregions: The suprapyramidal (upper/inner) blade (sDG) that is located adjacent to the hippocampal fissure, and the infrapyramidal (lower/outer) blade (iDG) (Amaral and Witter 1989) (Figure 1A). In vitro examinations of granule cell properties suggest heterogeneous membrane and action potential (AP) properties are evident within each of the two blades (Mishra and Narayanan 2020). To what extent information processing at the level of synaptic plasticity differs between the two blades is unclear. However, distinctions between the anatomical inputs to sDG and iDG suggest that this might be the case. The primary information source for the DG is the perforant path (PP) that delivers information from the entorhinal cortex (EC) (Hjorth‐Simonsen 1972; Steward 1976). The PP is segregated into two distinct afferent pathways: the medial PP (MPP) that transmits information from the medial EC and synapses at the level of the middle third of the molecular layer, and the lateral PP (LPP) that carries information from the lateral EC and synapses at the outer third of the molecular layer (Hjorth‐Simonsen 1972; Steward 1976; Groen, Kadish, and Wyss 2002). It is believed that these pathways provide functionally distinct information to the hippocampus, whereby the MPP delivers information about the spatial characteristics of sensory experience, and the LPP delivers information about nonspatial characteristics such as item identity (Deshmukh and Knierim 2011; Fyhn et al. 2004; Hargreaves et al. 2005; Kerr et al. 2007; Young et al. 1997).

Anatomical scrutiny has indicated that the LPP projects preferentially to the sDG and that the MPP projects preferentially to the iDG (Tamamaki 1997; Wyss 1981). The functional consequence may be that the sDG mainly processes nonspatial, and the iDG predominantly processes spatial information. Proof in support of this prediction is controversial. Although evidence does in fact exist that the sDG and iDG may fulfill different tasks in terms of information processing and storage, scrutiny of experience‐dependent expression of immediate early genes in neuronal nuclei, after novel exposure to environments of different spatial‐/nonspatial contexts, suggests that the iDG may be more concerned with the processing of item‐place experience and landmark‐based directional navigation (Hoang, Aliane, and Manahan‐Vaughan 2018). The sDG, by contrast, may play a role in the processing of spatial navigation in the absence of ostensible landmarks (Chawla et al. 2005), suggesting that both structures contribute to spatial information processing.

Experience‐dependent information storage within the hippocampus is mediated by persistent plasticity processes, such as changes in intrinsic excitability (Lopez‐Rojas, Heine, and Kreutz 2016; Mishra and Narayanan 2022) and synaptic plasticity (Abraham, Jones, and Glanzman 2019; Bliss and Lomo 1973; Hagena and Manahan‐Vaughan 2024), which are supported by dynamic responses in, for example, interneurons (Tzilivaki et al. 2023), astrocytes (Jourdain et al. 2007; Wenzel et al. 1991) and oligodendrocytes (Kol and Goshen 2021; Yamazaki et al. 2014). DG granule cells of the suprapyramidal blade are believed to comprise a substrate for memory encoding (Josselyn and Tonegawa 2020). This process is likely to be promoted by hippocampal synaptic plasticity that can persist for days and weeks in behaving animals (Hagena and Manahan‐Vaughan 2024). Studies of putative engram cells in the DG have focused on the suprapyramidal blade (Josselyn and Tonegawa 2020), or hilus (Deng, Mayford, and Gage 2013), with the role of the infrapyramidal blade unclear as yet. However, differences in the experience‐dependent activation of granule cells in the infra‐and suprapyramidal blades of the dorsal DG have been reported (Hoang, Aliane, and Manahan‐Vaughan 2018), and depending on the experience, the infrapyramidal blade shows lower responsiveness than the upper blade (Chawla et al. 2005; Erwin et al. 2020). Furthermore, although a multitude of studies have scrutinized the frequency dependency and response characteristics of synaptic plasticity in the dorsal DG, to our knowledge, these studies have focused almost exclusively on the sDG (Altinbilek and Manahan‐Vaughan 2009; Frey and Frey 2009; Kenney and Manahan‐Vaughan 2013; Krug et al. 1983). To what extent the iDG expresses frequency‐dependent synaptic plasticity in vivo has been largely unexplored.

The aim of the present study was therefore to compare the frequency dependency of synaptic plasticity in dorsal sDG and iDG in freely behaving adult rats. We chose to analyze synaptic plasticity enabled by the MPP as this is the best studied pathway to the dorsal DG. Given the importance of the N‐methyl‐D‐aspartate receptor (NMDAR) for hippocampal synaptic plasticity (Kauer, Malenka, and Nicoll 1988; Mody and Heinemann 1987; Thiels et al. 1996), the expression of GluN1, GluN2A, and GluN2B were also scrutinized. Furthermore, using patch clamp recordings in acute brain slices, we assessed to what extent intrinsic properties of mature granule cells differ in sDG and iDG. We report here that synaptic plasticity in the iDG is weaker, less persistent and less responsive to afferent frequencies than synaptic plasticity in the sDG in vivo. Moreover, AP properties and firing frequency differ between blades and all three examined GluN subunits exhibit significantly lower expression in the iDG. These differences are likely to potently influence the nature and content of information processing in the iDG.

## 2 Materials and Methods

### 2.1 Subjects

The study was carried out in accordance with the European Communities Council Directive of September 22, 2010 (2010/63/EU) for care of laboratory animals. All experiments were conducted according to the guidelines of the German Animal Protection Law and were approved in advance by the North Rhine‐Westphalia State Authority (Bezirksamt, Arnsberg). The 3R strategy was implemented to reduce the number of animals included in experimentation.

For all experiments, male Wistar rats (Charles River, Sulzfeld, Germany) were used to reduce variability in plasticity responses due to the estrous cycle (Warren et al. 1995) and thereby keep animal numbers lower. Animals were maintained on a 12 h light/12 h dark cycle in temperature and humidity monitoring cabinets and had *ad libitum* access to water and food. Animals were not exposed to an enriched environment before, or during experimental procedures. For in vivo experiments, animals were housed individually after surgical implantation of electrodes.

### 2.2 Surgery

Animals (7–8 weeks old at the time of surgery) underwent chronic implantation of electrodes in the dorsal DG, (Manahan‐Vaughan 2018). Briefly, electrodes were self‐made from polyurethane‐coated stainless‐steel wire (diameter: 127 μm, Biomedical Instruments, Zöllnitz, Germany) and screws connected to silver‐coated copper wire served as ground and reference electrodes. Anesthesia was implemented using sodium pentobarbital applied intraperitoneally (i.p.) (Nembutal, 52 mg/kg, Boehringer Ingelheim, Ingelheim, Germany). One monopolar recording electrode was placed in the sDG (ca. 3.1 mm posterior to bregma, ca. 1.9 mm lateral from midline, 3.9–4.4 mm below the lower edge of the skull) and one in the iDG (ca. 1.9 mm posterior to bregma, ca. 1.0 mm lateral from midline, ca. 5 mm below the skull) according to a stereotaxic rat brain atlas (Paxinos and Watson 2014). A bipolar stimulation electrode was positioned in the MPP (6.9 mm posterior to bregma, 4.1 mm lateral from midline, 3.8–4.2 mm below the skull). After the evoked response in both blades remained stable, the electrode assembly was sealed and fixed to the skull with dental acrylic (Paladur, Heraeus Kulzer GmbH, Hanau, Germany). Pre‐ and postsurgical analgesia was implemented using meloxicam in solution (0.2 mg/kg, i.p.; Metacam, Boehringer Ingelheim Vetmedica GmbH, Ingelheim, Germany).

### 2.3 Electrophysiological Recordings

Recording of evoked responses in sDG and iDG were commenced no sooner than 10 days after surgery. Evoked responses in the sDG and iDG were evoked by stimulating the MPP at a low frequency (0.025 Hz) using single biphasic square wave pulses (0.2 ms duration per half wave). For all responses, the amplitude of the PS (population spike) and the slope of the field excitatory postsynaptic potentials (fEPSP) were measured.

Before the first experiment, rats were habituated overnight to the recording chamber (40 × 40 × 50 cm). They could move freely within the recording chamber during the experiments. Disturbances of the animals were kept to an absolute minimum. The stimulus–response (input–output [I/O]) relationship (100–900 μA, in 100 μA steps) was recorded to determine the maximum PS. This range of stimulus intensities was chosen to determine the range of potential amplitudes that an individual animal can generate. Here, it is also important to note that although granule cells of the DG are subjected to strong inhibitory control (Houser 2007; Jinde, Zsiros, and Nakazawa 2013), under specific circumstances they are capable of generating potentials that are multiples of hundreds of percent larger than those evoked by pretreatment test pulses. For example, treatment of freely behaving rats with fucose results in evoked potentials are 300% larger than those evoked by test pulses and which endure for days (Krug et al. 1994), LTP at LPP‐DG synapses of 800% has been reported (Vyleta and Snyder 2021) and others have shown that stimulation of the locus coeruleus in urethane‐anesthetized rats can result in enhancements of the PS in the DG of up to 200% (Edison and Harley 2012) even though high‐frequency stimulation (HFS) under urethane anesthesia substantially reduces the magnitude of potentiation in the DG (Cain, Boon, and Hargreaves 1992). Findings such as these tell us that DG granule cells have a huge capacity to engage in potentiation (or enhanced physiological) responses. It is therefore important to ascertain the range of responses possible during electrophysiological stimulation by means of test‐pulses at different stimulation intensities, as is conducted during an I/O response determination. This is crucial when subsequently scrutinizing for synaptic depression: a test‐pulse stimulus intensity that is too low within the range of fEPSP responses will mean that an LTD effect cannot be detected even though the synapses may be capable of engaging in LTD. If one is studying bidirectional synaptic plasticity a test‐pulse intensity must be used that allows room to detect both synaptic depression and potentiation.

For the remainder of the experiment, a stimulus intensity producing ca. 40% of the maximum I/O response was used to evoke responses. For each time‐point, five evoked responses were averaged. The first six time‐points were recorded at 5 min intervals and these values served as a reference: All data points were calculated as a percentage of the mean of these first six time‐points. After recording three further time‐points at 5 min intervals, the recording interval was extended to 15 min and 15 additional time‐points were then recorded. On the next morning, one additional hour of recordings was performed. Rats exhibiting stable evoked responses during a control (test‐pulse stimulation) experiment were used for further experiments to assess synaptic plasticity responses. In this case, experiments were always spaced by at least 7 days and the pre‐experiment I/O response was used to verify that no residual effects on evoked potentials were retained from previous experiments.

### 2.4 Patterned Afferent Stimulation Protocols

Afferent frequencies protocols were used that are known to induce synaptic plasticity in the sDG of behaving rats (Jeffery and Morris 1993; Manahan‐Vaughan 2018; Schulz et al. 1999). Low‐frequency stimulation (LFS) at 1 Hz was applied as 900 consecutive pulses. LFS at 10 Hz (450 consecutive pulses) and 5 Hz (10 bursts of 20 pulses with an inter‐burst interval of 5 s) were also applied. HFS was applied at 200 Hz and 400 Hz, as these frequencies have been reported to generate very robust forms of LTP in the DG of behaving rats (Jeffery and Morris 1993; Schulz et al. 1999). Both protocols consisted of 10 bursts of each 15 pulses, applied either at 200 Hz or 400 Hz, with an inter‐burst interval of 10 s. Recordings of evoked responses were halted during patterned afferent stimulation and recommenced 5 min after the stimulation protocol had concluded.

### 2.5 Verification of Electrode Positions

At the end of the study, brains were removed for histological verification of the position of the electrodes (Figure 1B). Coronal sections (30 μm thick) were stained in 0.1% cresyl violet. Animals with suboptimally implanted electrodes were excluded from data analysis. In some animals, one of the two recording electrodes was implanted incorrectly, but the data was analyzed for the correctly positioned electrode: therefore, the animal numbers are not equal for all experimental conditions.

### 2.6 Patch Clamp Recordings

For acute brain slice preparations, rats (7–9 weeks old) were euthanized by isoflurane anesthesia and decapitation, followed by rapid brain extraction. Horizontal slices (350 μm) were prepared in ice cold, oxygenated sucrose solution (in mM: 87 NaCl, 2.6 MgSO_4_, 75 sucrose, 2.5 KCl, 1.25 NaH_2_PO_4_, 26 NaHCO_3_, 0.5 CaCl_2_, 2 D‐Glucose) using a vibratome. Slices were incubated at 35°C with oxygenated artificial cerebrospinal fluid (aCSF; in mM: 125 NaCl, 3 KCl, 2.5 CaCl_2_, 1.3 MgSO_4_, 1.25 NaH_2_PO_4_, 26 NaHCO_3_, 13 D‐Glucose) for at least 30 min.

Patch clamp recordings were performed as previously described (Sudkamp, Shchyglo, and Manahan‐Vaughan 2024). To obtain recordings no channel, transporter, pump, or receptor blockers were used thus, any remaining currents contributed to the measurements. Briefly, slices were continuously perfused with oxygenated aCSF (1–2 mL/min) in a recording chamber under an upright microscope and bath temperature maintained at 32 to 34°C to ensure stable control of oxygenation and pH without affecting intrinsic electrophysiological properties of the neurons (Huang and Uusisaari 2013). Borosilicate glass recording pipettes (resistance: 4‐7 MΩ) were filled with an intracellular solution (in mM: 97.5 potassium gluconate, 32.5 KCl, 5 EGTA, 10 Hepes, 1 MgCl_2_, 4 Na_2_ ATP; at pH 7.3). With this pipette solution, chloride reversal currents depolarize (33°C: E(Cl−) = −35.6 mV) at resting membrane potential. Recordings were conducted from visually identified somata of mature granule cells in the middle to deep granule cell layer of the sDG and iDG (close to the molecular layer) to minimize the chance of recordings from adult‐born (immature) granule cells that are mostly located in the inner granule cell layer (Figure 4 A‐C). Intrinsic membrane properties were recorded with an amplifier using the PATCHMASTER acquisition software (HEKA Elektronik GmbH, Lambrecht/Pfalz, Germany). After low‐pass filtering (2.9 kHz), data were digitized at 10 kHz.

After recordings were complete, patched cells were filled with biocytin (1 mg/mL, Sigma‐Aldrich, St. Louis, USA) and slices were fixed in 4% paraformaldehyde (PFA) in phosphate buffered saline (PBS) and then kept in 30% sucrose solution for at least 7 days, before being cut into slices of 60 μm thickness at −35°C. Biocytin‐filled granule cells were detected using Streptavidin Cy3 (1:1000; Dianova, Hamburg, Germany) and nuclei were visualized using 4′,6‐diamidino‐2‐phenylindole (DAPI) in mounting medium (SCR‐038448; Dianova, Hamburg, Germany).

### 2.7 Immunohistochemistry

Immunohistochemistry was performed to assess the expression of the GluN1, GluN2A, and GluN2B subunits of the NMDAR, as previously described (Collitti‐Klausnitzer et al. 2021). Briefly, euthanized animals were transcardially perfused with Ringer's solution containing heparin (0.2%), followed by 4% PFA in PBS. After removal, brains were fixed in 4% PFA followed by 30% sucrose. Horizontal sections (30 μm thick) were cut, and for each animal, three sections including the hippocampus (ca. 3.6–4.1 mm ventral to bregma) were used for immunohistochemistry (Figure 1A). After pretreatment in 0.3% H_2_O_2_, sections were rinsed and incubated with blocking solution containing normal serum (10%), avidin (20%) in PBS with 0.2% Triton X‐100 (PBS‐Tx). Sections were incubated overnight in primary antibody solution with goat polyclonal anti‐NMDAε2 (GluN2B) (1:250; sc‐1469, Santa Cruz Biotechnology, Santa Cruz, USA) in 0.2% PBS‐Tx with 1% normal serum and 20% biotin. After washing, sections were incubated with biotinylated horse anti‐goat (1:500; BA‐9500, Vector Laboratories, Burlingame, USA) antibody in 0.1% PBS‐Tx with 1% normal serum. Sections were incubated with an avidin‐biotin complex (ABC) kit (PK‐6100, Vector Laboratories, Burlingame, USA) in 0.1% PBS‐Tx with 1% normal serum, after washing. An additional amplification step was performed for other receptors. For this, sections were incubated with primary antibody solutions containing mouse monoclonal anti‐NMDAR1 (GluN1) (1:200; 556308, PharMingen, Becton, Dickinson and Company, Frankline Lakes, USA) or rabbit polyclonal anti‐NMDAε1 (GluN2A) (1:750; sc‐9056, Santa Cruz Biotechnology, Santa Cruz, USA), in tris‐buffered saline containing 0.2% Triton X‐100 and 1% BSA, for 5 days at 4°C. After washing, incubation in biotinylated horse anti‐mouse (BA‐2001, Vector Laboratories, Burlingame, USA), or goat anti‐rabbit (BA‐1000, Vector Laboratories, Burlingame, USA), and further washing, sections were incubated with an ABC reaction. This was followed by the amplification with a biotinylated tyramide solution followed by another ABC reaction. After final washing all sections were treated with diaminobenzidine (DAB) and 0.01% H_2_O_2_.

### 2.8 Data Processing and Statistics

#### 2.8.1 In Vivo Electrophysiological Recordings

For in vivo electrophysiological experiments, data were expressed as a mean percentage of the average reference value, and the mean percentage ± SEM of all animals of each group was visualized. Supplementary Figure 1 reports the individual responses of each animal for each timepoint recorded in each experiment. To evaluate the I/O relationship, PS and fEPSP values were visualized as mean ± SEM of all animals in which electrodes in sDG and iDG were implanted correctly. The latency from the artifact of the stimulus pulse to the start of the fEPSP (lat 1) and to the start of the PS (lat 2), was calculated as the mean of all I/O relationship responses from either iDG or sDG recordings for each animal. Latencies for sDG and iDG were coherent with latencies detected in the MPP‐DG pathway (Abraham, Bliss, and Goddard 1985). Data were visualized as difference between the two blades (Δ latency iDG‐sDG).

Using Statistica software (StatSoft. Inc., Tulsa, OK, USA) analysis of variance (ANOVA) with repeated measures was conducted to analyze differences between patterned stimulation and test‐pulse stimulation, or to identify differences in effects of the same patterned stimulation protocol on sDG and iDG. Besides *p*‐values, η_p_^2^ are reported and indicate a large effect size for significant results. Latencies were statistically evaluated using a dependent *t* test after testing for normal distribution.

#### 2.8.2 Patch Clamp Recordings

Patch clamp data were analyzed offline using FITMASTER software (HEKA Elektronik GmbH) and AP feature software (MATLAB code provided by Prof. M. Volgushev, Department of Psychology, University of Connecticut). Data processing was performed according to established procedures (e.g., Sudkamp, Shchyglo, and Manahan‐Vaughan 2024): During recordings, the cells were maintained at their resting membrane potential. By applying square current pulses (5 pA steps, duration 600 ms) the first evoked AP was used to analyze the following properties: AP threshold (mV), spike amplitude from threshold (mV), peak amplitude (mV), afterhyperpolarization (AHP) depth (mV) measured from the AP threshold, AHP minimum (mV), total spike time (ms) from AP onset to the AHP minimum, width of the AP half‐amplitude (ms), time from AP onset to peak (ms), and the time from peak to AHP (ms). In addition, passive and active neuronal properties were assessed comprising resting membrane potential (mV); input resistance (MOhm); membrane time constant (tau, in ms); and excitatory threshold (pA). The resting membrane potential was determined from the mean of a 30 s baseline recording. The input resistance was calculated from the slope of the linear fit of the relationship between the change in membrane potential and the intensity of the injected current (−60 to 20 pA). Tau was determined from an exponential fit of the averaged voltage decay. The minimum current necessary to evoke an AP from the resting potential was defined as excitatory threshold. Square current pulses (1 s duration) through the patch‐clamp electrode (50–700 pA, 50 pA steps) were applied to examine firing properties. The firing frequency was calculated from the number of spikes elicited during the application of each current step. Spike frequency adaptation was calculated by counting the total number of spikes during each 100 ms of the 1 s square current pulse of 400 pA, this number was converted into frequency. Cells of each blade, that entered depolarization block (DB) with increasing current steps, were evaluated in more detail: resting membrane potential (mV), mean plateau of the DB (mV), and the minimum current step (pA) when DBs (duration at least 250 ms) is first developed were analyzed. The proportion of cells showing persistent firing (PF) and DB was calculated for each blade.

Data of patch clamp recordings were visualized using scatter plots including the median ± interquartile range of all cells of each blade. After evaluation of normal distribution (Kolmogorov–Smirnov test), differences in active and passive neuronal and AP properties, between granule cells of both blades, were all examined using a Mann–Whitney *U* test. Proportions of cells exhibiting DB and PF were compared using a chi square test. The results for the firing frequency and spike frequency adaptation were visualized as mean ± SEM of all cells of each blade and were compared using repeated measures ANOVA.

#### 2.8.3 Immunohistochemistry

Using a light microscope (Leica DMR, Wetzlar, Germany) with a digital camera (MBF Bioscience, Williston, Vermont, USA), photomicrographs of stained sections were acquired and stored in TIFF format to analyze receptor expression. ROIs (regions of interest) of the middle molecular layer of each blade of the DG were analyzed at 2.53 lens magnification. Pictures were obtained using Neurolucida software (MBF Bioscience, Williston, Vermont, USA) and quantified using open‐source ImageJ software (National Institute of Health, USA). For deconvolution of the color information and conversion to 8‐bit format, the “Color Deconvolution” plugin in ImageJ was used. Background values from fimbria and deep cerebral white matter tracts were averaged and subtracted from value within the ROI of the corresponding images. For scaling of data from several independent staining/plates, a generalized residual sum of squares algorithm to account for batch effects of staining intensities in R software was used (Kreutz et al. 2007; Heyde et al. 2014). These preprocessed values were used for further analysis of immunohistochemistry data: The mean of these values of three sections was calculated for each animal and graphs were created that represent the mean of all animals ± SEM. For statistical analysis, data were assessed for normal distribution (Kolmogorov–Smirnov test) and an unpaired *t* test was performed to compare expression pattern in sDG and iDG for each NMDAR subunit in the middle, outer and inner compartment of the molecular layer and the granule cell layer using GraphPad Prism software (version 6, GraphPad Software Inc., USA). The expression patterns of NMDAR subunits of the middle and outer molecular layer of sDG were published previously (Collitti‐Klausnitzer et al. 2021).

For all data, the level of significance was set to *p* < 0.05. “N” corresponds to the number of animals and “n” describes the number of cells examined in patch clamp recordings.

## 3 Results

### 3.1 Maximal Neuronal Responsiveness in the Infrapyramidal Blade Is Lower Compared to the Suprapyramidal Blade of the Dentate Gyrus

To compare synaptic plasticity in sDG and iDG, animals were implanted with one recording electrode in sDG and one in iDG (Figure 1B), to enable examination of the effect of test‐pulse stimulation and patterned stimulation of the MPP on responses evoked simultaneously in both blades of the DG.

For comparison of I/O relationships, only those animals were used where both recording electrodes were positioned correctly (Figure 1C, *N* = 8). Here, it was found that the PS of evoked responses in the sDG were significantly different from the iDG (*F*_(1,14)_ = 6.928, *p* < 0.05, η_p_^2^ = 0.331), whereas fEPSPs were not significantly different between the two blades (*F*_(1,14)_ = 3.149, *p* = 0.098). However, with increasing stimulus intensities PS and fEPSP of evoked responses increased in both blades, although this change was weaker in the iDG compared to the sDG (PS: *F*_(8,112)_ = 11.544, *p* < 0.0001, η_p_^2^ = 0.452; fEPSP: *F*_(8,112)_ = 4.963, *p* < 0.0001, η_p_^2^ = 0.262; Figure 1C). Post hoc analysis (Fisher's LSD test) revealed that the PS becomes significantly different between blades, starting at a stimulus intensity of 400 μA until the maximum intensity that was tested (900 μA), whereas the fEPSP of evoked responses was significantly different at intensities from 500 to 900 μA. An evaluation of latencies of the I/O relationship revealed no significant difference between iDG and sDG (Figure 1D).

**FIGURE 2:**
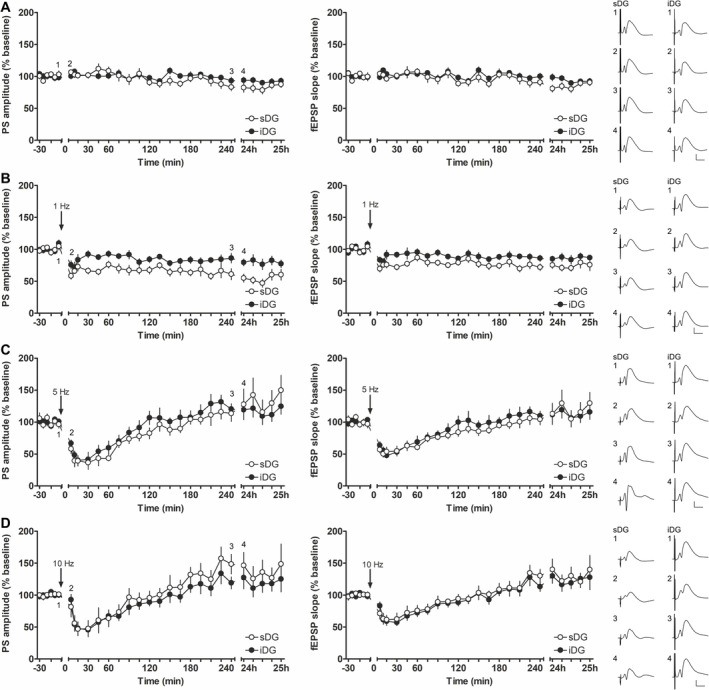
The magnitude of LTD induced by LFS at 1 Hz is weaker in the infrapyramidal compared to the suprapyramidal blade of the dentate gyrus. (A) Test‐pulse stimulation, without application of patterned afferent stimulation, evokes stable population spikes (PS) and field excitatory postsynaptic potentials (fEPSPs) recorded in the suprapyramidal (sDG, *N* = 10) and infrapyramidal blade (iDG, *N* = 13) over a period of 4.5 h, followed by one further hour of recordings 24 h after the experiment was started. Analog examples of evoked field potentials recorded in sDG and iDG at the time points (1) 5 min, (2) 5 min, (3) 240 min, and during (4) 24 h recording. (B) Low‐frequency stimulation (LFS) at 1 Hz results in long‐term depression (LTD) in sDG (*N* = 9) and iDG (*N* = 11). LTD induced in the sDG is significantly stronger compared to the iDG. Analog examples of evoked field potentials recorded in each blade at the time points (1) 5 min before, (2) 5 min, (3) 240 min and (4) 24 h after LFS at 1 Hz. (C) LFS at 5 Hz results in a similar degree of transient depression followed by a potentiation in sDG (*N* = 7) and iDG (*N* = 9). Representative examples of evoked field potentials recorded in sDG and iDG at the time points (1) 5 min before, (2) 5 min, (3) 240 min, and (4) 24 h after LFS at 5 Hz. (D) A transient depression followed by a potentiation of a similar magnitude was induced in sDG (*N* = 7) and iDG (*N* = 9) by LFS at 10 Hz applied to the medial perforant path. Representative analog examples of evoked field potentials recorded in each blade at the time points (1) 5 min before, (2) 5 min, (3) 240 min, and (4) 24 h after LFS at 10 Hz. (A–D) Calibration: Vertical bar: 5 mV, horizontal bar: 5 ms. Supplementary Figure 1 represents the values of each animal for each time point during the recording.

To examine if evoked responses remained stable, basal synaptic transmission was recorded over a 24 h period (4–5 h), followed by 60 min of recordings (at 15 min intervals) 1 day after the experiment began (Figure 2A, for data of individual animals see Supplementary Figure 1). Evoked responses in sDG (*N* = 10) and iDG (*N* = 13) remained stable over time and were not significantly different between the two blades (PS: *F*_(1,21)_ = 1.765, *p* = 0.198; fEPSP: *F*_(1,21)_ = 2.874, *p* = 0.105).

### 3.2 Low‐Frequency Stimulation at 1 Hz Induces Stronger LTD in the Suprapyramidal Blade Compared to the Infrapyramidal Blade

LFS at 1 Hz induces persistent (> 24 h) LTD in the sDG (Altinbilek and Manahan‐Vaughan 2009; Kenney and Manahan‐Vaughan 2013). Here, too, LFS at 1 Hz (Figure 2B) triggered LTD in the sDG (PS: *F*_(1,17)_ = 59.268; *p* < 0.0001, η_p_^2^ = 0.77; fEPSP: *F*_(1,17)_ = 25.362; *p* < 0.001, η_p_^2^ = 0.599 versus test‐pulse stimulation only). LTD was also induced by 1 Hz (900 pulses) in the iDG (PS: *F*_(1,22)_ = 16.342; *p* < 0.001, η_p_^2^ = 0.426; fEPSP: *F*_(1,22)_ = 9.829; *p* < 0.01, η_p_^2^ = 0.309). A closer comparison of evoked responses of both blades after LFS at 1 Hz (Figure 2B), revealed that LTD in the sDG (*N* = 9) was stronger than in the iDG (*N* = 11) (PS: *F*_(1,18)_ = 17.985; *p* < 0.001, η_p_^2^ = 0.5; fEPSP: *F*_(1,18)_ = 7.234; *p* < 0.05, η_p_^2^ = 0.287), indicating that LTD in the blades of the dorsal DG differs in magnitude in response to 1 Hz stimulation.

### 3.3 Afferent Stimulation at 5 or 10 Hz Induces Transient Depression Followed by Slow Potentiation in the Supra‐ and Infrapyramidal Blade

Afferent frequencies of 5 or 10 Hz generate transient depression followed by a slowly developing potentiation in MPP‐sDG synapses (Collitti‐Klausnitzer et al. 2021; Twarkowski, Hagena, and Manahan‐Vaughan 2016): Here too, both frequencies evoked a short initial depression followed by a later potentiation in both the sDG (*N* = 7, each) and iDG (*N* = 9, each). Evoked responses after 5 Hz (Figure 2C) and 10 Hz (Figure 2D) were not significantly different between both blades (5 Hz: PS: *F*_(1,14)_ = 0.265; *p* = 0.615; fEPSP: *F*_(1,14)_ = 0.247; *p* = 0.627; 10 Hz: PS: *F*_(1,14)_ = 0.589; *p* = 0.455; fEPSP: *F*_(1,14)_ = 0.216; *p* = 0.649). In comparison with baseline experiments conducted using test‐pulse stimulation alone, no significant change was detected for either LFS at 5 Hz (fEPSP: *F*_(1,15)_ = 3.233; *p* = 0.092, PS: *F*_(1,15)_ = 0.809; p = 0. 383), or LFS at 10 Hz (PS: *F*_(1,15)_ = 1.218; *p* = 0.287; fEPSP: *F*_(1,15)_ = 0.820; *p* = 0.38). For the iDG a comparison with baseline experiments revealed no significant difference for LFS at 5 Hz (PS: *F*_(1,20)_ = 0.458; *p* = 0.506; fEPSP: *F*_(1,20)_ = 2.084; *p* = 0.164) and at 10 Hz (PS: *F*_(1,20)_ = 0.325; *p* = 0.575; fEPSP: *F*_(1,20)_ = 0.521; *p* = 0.479). However, assessment of sDG and iDG responses to 5 and 10 Hz stimulation, compared to test‐pulse (baseline) evoked responses, all revealed a significant difference in the interaction effect of time × group (Supplementary Table 1). In general, stimulation of the MPP at 5 or 10 Hz evoked equivalent responses in sDG and iDG.

### 3.4 LTP Induced by HFS at 200 and 400 Hz Evoke LTP of Greater Magnitudes in the Suprapyramidal Blade Compared to the Infrapyramidal Blade

**FIGURE 3:**
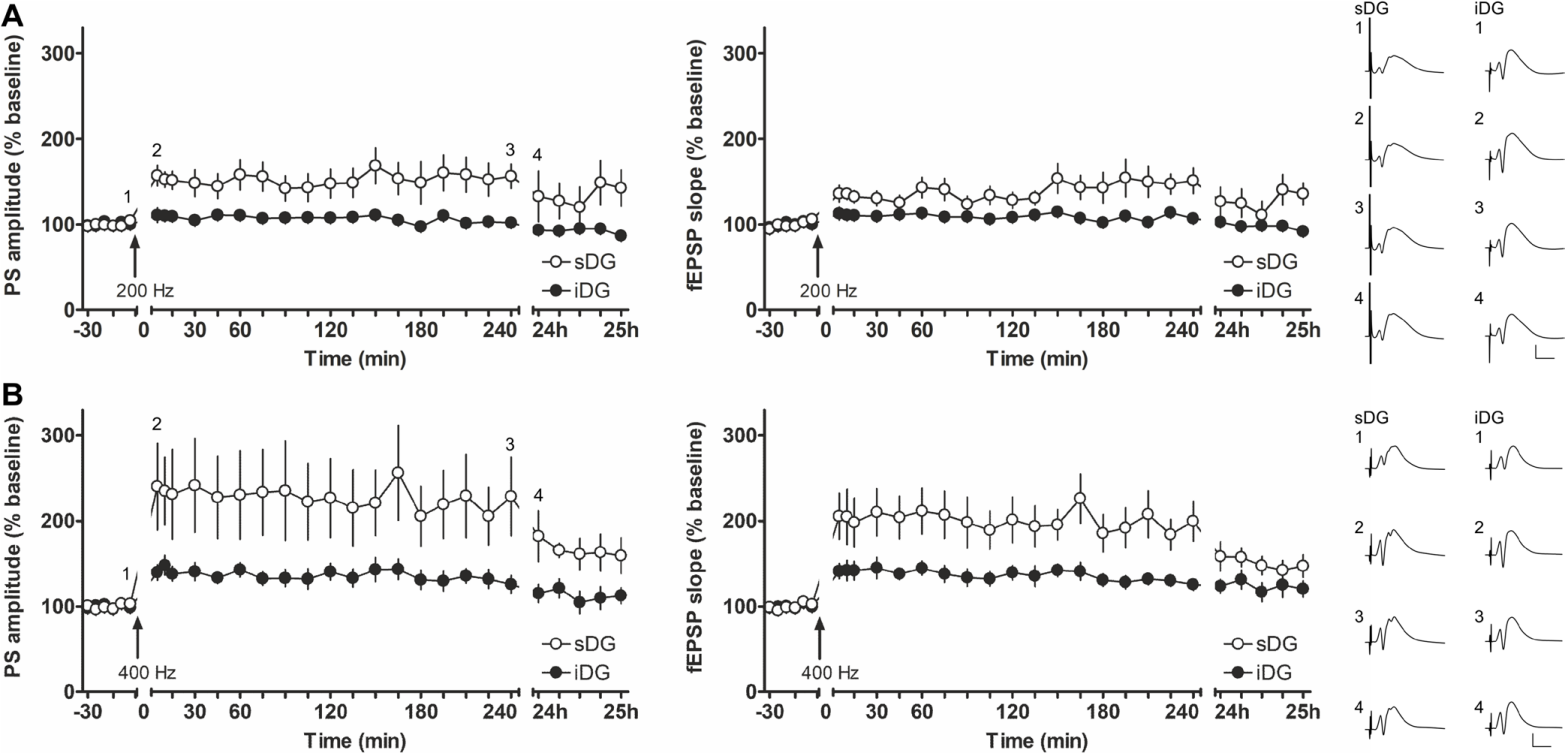
In the infrapyramidal blade, HFS at 200 Hz is ineffective in changing synaptic transmission and 400 Hz HFS induces LTP of a smaller magnitude compared to LTP induced in suprapyramidal blade. (A) HFS at 200 Hz results in LTP in the suprapyramidal blade (sDG, *N* = 9) that is significantly different from the unchanged synaptic response detected in the infrapyramidal blade (iDG) after 200Hz HFS (*N* = 12). Analog examples of evoked field potentials recorded in the sDG and iDG at the time points (1) 5 min before, (2) 5 min, (3) 240 min, and (4) 24 h after HFS at 200 Hz. (B) LTP induced by HFS at 400 Hz of the medial perforant path induces a weaker LTP in the iDG (*N* = 11) compared to the significantly stronger LTP induced in the sDG (*N* = 8). Representative examples of evoked field potentials recorded in the sDG and iDG at the time points (1) 5 min before, (2) 5 min, (3) 240 min, and (4) 24 h after HFS at 400 Hz. (A, B) Calibration: Vertical bar: 5 mV, horizontal bar: 5 ms. Supplementary Figure 1 represents the values of each animal for each time point during the recording.

HFS of the MPP at 200 Hz induced LTP that lasted over 24 h in MPP‐sDG synapses (*N* = 9, Figure 3) compared to test‐pulse stimulation (PS: *F*_(1,17)_ = 13.788; *p* = 0.01, η_p_^2^ = 0.448; fEPSP: *F*_(1,17)_ = 21.314; *p* = 0.001, η_p_^2^ = 0.556). By contrast 200 Hz HFS elicited no significant change in evoked responses in the iDG (*N* = 12) in comparison to test‐pulse stimulation (PS: *F*_(1,23)_ = 1.462; *p* = 0.239; fEPSP: *F*_(1,23)_ = 3.368; *p* = 0.079). Evoked responses were also significantly different after 200 Hz HFS (Figure 3A) when both blades were compared (PS: *F*_(1,19)_ = 10.623; *p* < 0.01, η_p_^2^ = 0.359; fEPSP: *F*_(1,19)_ = 11.796; *p* < 0.01, η_p_^2^ = 0.383).

Increasing the afferent frequency to 400 Hz resulted in LTP in iDG synapses (*N* = 11) compared to test‐pulse stimulation (PS: *F*_(1,22)_ = 18.922; p = 0.001, η_p_^2^ = 0.462; fEPSP: *F*_(1,22)_ = 22.932; *p* < 0.0001, η_p_^2^ = 0.510). This LTP was smaller in magnitude than responses induced by 400 Hz HFS in the sDG (*N* = 8) in comparison to test‐pulse stimulation (PS: *F*_(1,16)_ = 11.793; *p* < 0.01, η_p_^2^ = 0.424; fEPSP: *F*_(1,16)_ = 28.981; *p* < 0.0001, η_p_^2^ = 0.644). Comparison of the LTP responses in both blades (Figure 3B) confirmed that LTP in iDG was significantly smaller than LTP in sDG (PS: *F*_(1,17)_ = 5.843; *p* < 0.05, η_p_^2^ = 0.256; fEPSP: *F*_(1,17)_ = 8.944; *p* < 0.01, η_p_^2^ = 0.345).

Overall, these results suggest that synaptic depression, as well as synaptic potentiation, evoked by patterned afferent stimulation of the PP are substantially weaker in the iDG. Which leads to the question as to whether these differences may arise from blade‐specific granule cell properties and/or differences in the expression of plasticity‐related receptors in the two blades.

### 3.5 Distinct Action Potential Properties Differ Between the Supra‐ and Infrapyramidal Blades

**FIGURE 4:**
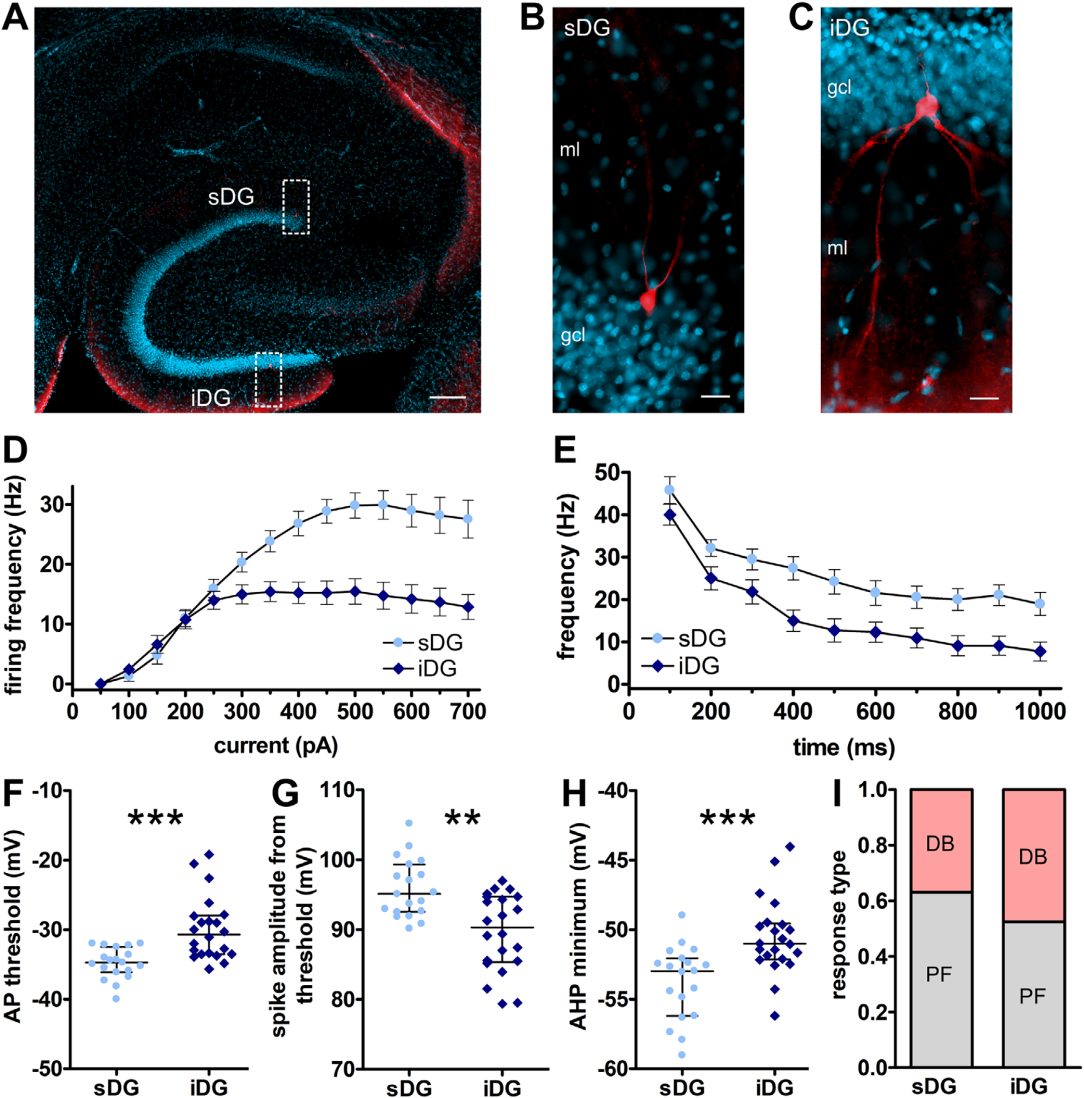
In vitro examination of the firing frequency and action potential properties exhibits differences between granule cells of the supra‐ and infrapyramidal blade of the dentate gyrus. (A–C) Biocytin filled granule cells in the blades of the dentate gyrus. (A) Example showing the location of patch clamp recordings in a horizontal section of the hippocampus. Granule cells in the sDG and iDG were examined and filled with biocytin (red). Nuclei are counterstained with DAPI (blue). Calibration: 200 μm. (B, C) Enlarged images of example in (A) of biocytin filled granule cells in (B) sDG and (C) iDG. Calibration: 20 μm. gcl: granule cell layer. ml: molecular layer. (D) The firing frequency differs between granule cells of the infrapyramidal (iDG) and suprapyramidal (sDG) blade. In granule cells of the iDG (*n* = 22, *N* = 6) the firing frequency is significantly smaller compared to granule cells recorded in the sDG (*n* = 19, *N* = 6). (E) Spike frequency adaptation at 400 pA was significantly faster in granule cells of iDG (*n* = 22, *N* = 6) compared to sDG (*n* = 19, *N* = 6) over the course of the 1 s current step. (F–H) Specific action potential properties differ in granules cells of the sDG (*n* = 19, *N* = 6) and iDG (*n* = 22, *N* = 6). (F) The threshold to induce an action potential is significantly higher in the iDG compared to the sDG. (G) The amplitude of the action potential from the threshold is significantly smaller in the iDG compared to the sDG. (H) The minimum of the afterhyperpolarization (AHP) is significantly lower in the sDG compared to the iDG. (I) In sDG, the largest proportion of cells show persistent firing (PF) during all current steps up to 700 pA, whereas in iDG the proportions of cells showing PF and cells developing depolarization block (DB) are nearly similar. In iDG 10 cells (of 21) and in sDG 7 cells (of 19) developed depolarization block with increasing current steps. (F–H) Significant differences are marked with asterisks: **p < 0.01; ***p < 0.001. Active and passive membrane and further action potential properties are shown in Supplementary Figure 2. Supplementary Figure 3 contains representative traces for D and E, as well as properties of cells developing DB.

Scrutiny of granule cell properties in the slice preparation, by means of patch clamp, revealed no difference between blades for all basic passive and active neuronal properties (Table 1, for individual data points see Supplementary Figure 2A–D; *N* = 6 each, *n* = 19 sDG, *n* = 22 iDG). However, the firing frequency differed significantly between blades (100–700 pA: *F*_(1,39)_ = 17.108; *p* < 0.001, η_p_^2^ = 0.305; Figure 4D, for individual data points see Supplementary Figure 3A,B). Starting from 350 pA, granule cells in the iDG exhibited a significantly smaller firing frequency compared to granule cells of the sDG (post hoc: Fisher's LSD test) demonstrating that the maximal frequency to fire APs differs between granule cells in both blades. A more rapid spike frequency adaptation (Figure 4E) was observed in iDG compared to sDG over 100 ms steps during the 1 s current injection at 400 pA (*F*_(1,39)_ = 10.398; *p* < 0.01, η_p_^2^ = 0.210).

**TABLE 1:** Summary of statistical analysis of basic membrane and action potential properties.

	*U*	*Z*	*p* value
**Membrane properties**			
Input resistance	179.0	0.771	0.441
Resting potential	174.0	0.902	0.367
Threshold	191.5	−0.444	0.657
Membrane time constant	186.0	0.588	0.556
**Action potential properties**			
Threshold	61.0	3.856	**< 0.001**
Spike amplitude from threshold	89.5	−3.111	**< 0.01**
AHP minimum	73.5	3.529	**< 0.001**
Peak amplitude	168.5	−1.046	0.296
AHP depth	161.5	1.229	0.219
Total spike time	199.5	0.235	0.814
Time to peak	142.0	1.739	0.082
Peak to AHP	187.0	−0.562	0.574
Width half amplitude	193.5	−0.392	0.695

*Note:* A Mann–Whitney *U* test was performed to assess differences in membrane and action potential properties in patch clamp recordings of acute brain slices between the infra‐ (iDG) and suprapyramidal (sDG) blades of the dentate gyrus (*N* = 6 each, *n* = 22 for iDG, *n* = 19 for sDG). Statistical significances are highlighted in bold font.

Moreover, examination of AP properties revealed differences between granule cells in the two blades (Figure 4F–H, Table 1). Here, the threshold to induce an AP (Figure 4F) was significantly higher in the iDG compared to the sDG (U = 61.0; Z = 3.856; *p* < 0.001), whereas the peak AP amplitude (Supplementary Figure 2E) did not differ between blades. However, the AP spike amplitude calculated from its threshold (Figure 4G) was significantly larger in the sDG compared to the iDG (U = 89.5; Z = −3.111; *p* < 0.01). Examination of the AHP, revealed that the minimum (Figure 4H) was significantly lower in the sDG compared to the iDG (U = 73.5; Z = 3.529; *p* < 0.001). By contrast the AHP depth was similar in both blades (Supplementary Figure 2F). All other examined AP properties, such as the total spike time, or the width half amplitude, were not significantly different between blades (Table 1, Supplementary Figure 2G–J).

With increasing current steps, to evaluate the firing frequency, we observed that some cells did not fire over the course of the 1 s recording period (Supplementary Figure 3B, iDG 700pA) suggesting that they enter a DB (Knauer and Yoshida 2019). This was detected in 7 cells of sDG and in 10 cells of iDG (Figure 4I), but the proportion of cells exhibiting PF or DB were not significantly different between blades (χ_(1)_^2^ test, *p* > 0.05). The lowest current step where a DB could be detected was significantly different between blades (U = 11.5; Z = 2.245; *p* < 0.05; Supplementary Figure 3C).

### 3.6 Expression of GluN1, GluN2A, and GluN2B Subunits Is Significantly Lower in the Middle Molecular Layer of the Infrapyramidal Compared to the Suprapyramidal Blade

**FIGURE 5:**
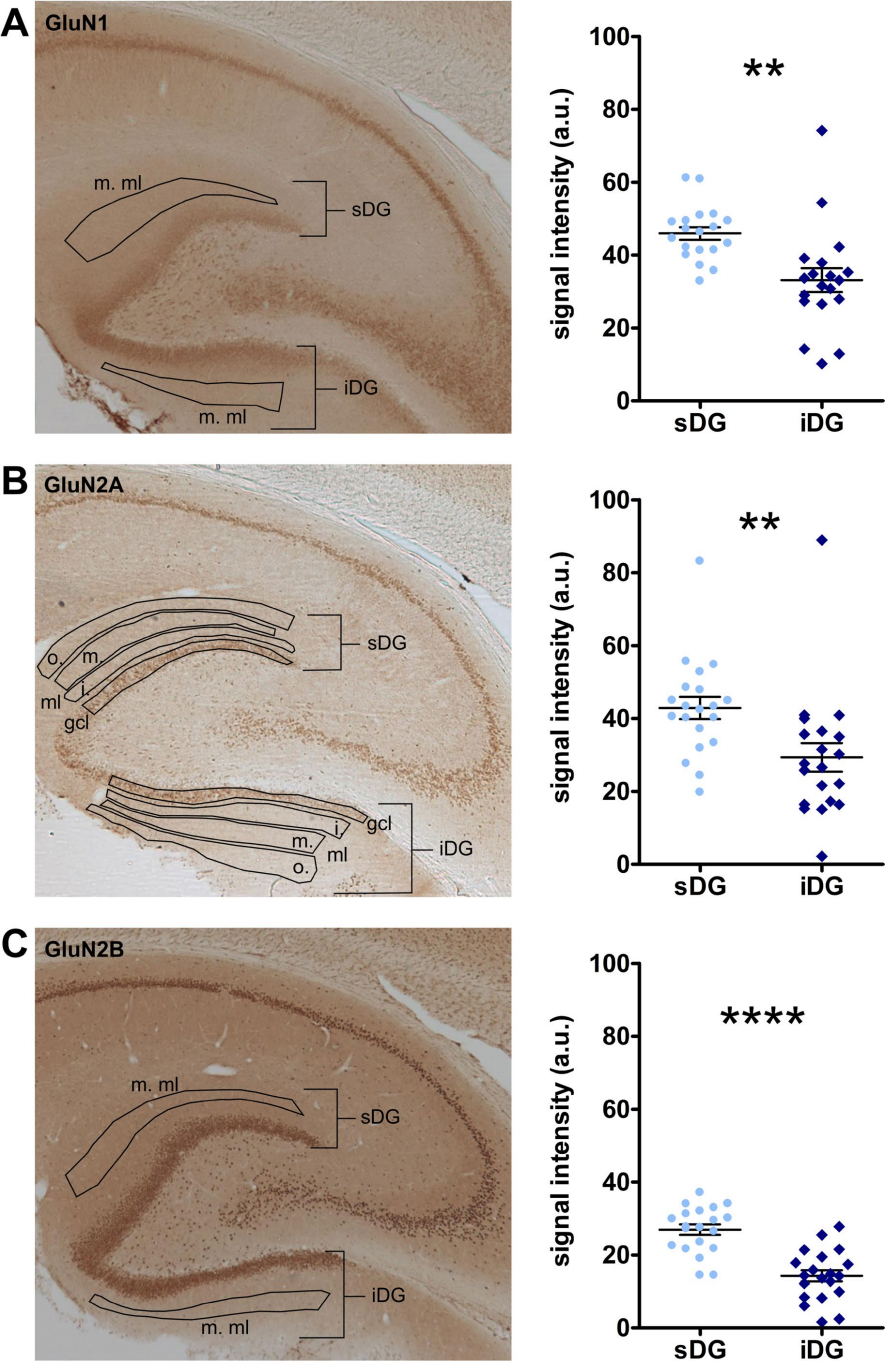
Expression of subunits of the NMDA receptor is lower in the middle molecular cell layer of the infrapyramidal compared to the suprapyramidal blade of the dentate gyrus. (A) Examination of GluN1 subunits in the middle molecular layer (m.ml) reveals a significantly lower expression in infrapyramidal (iDG) compared to the suprapyramidal (sDG) blade of the dentate gyrus (*N* = 19 each). Example of a DAB‐stained image of the hippocampal formation showing the expression of GluN1 subunit of the NMDA receptor. Outlines depict the regions of interest in the m.ml of the sDG and iDG. (B) GluN2A expression is significantly weaker in the m.ml of the iDG compared to the sDG (*N* = 20 each). Expression of the GluN2A subunit in the hippocampal formation shown in a representative DAB‐stained image. The regions of interest in the granule cell layer (gcl), outer (o.), inner (i.), and m. ml of sDG and iDG are depicted within the outlines. (C) In the m.ml of the iDG, GluN2B expression is significantly smaller than in the sDG (*N* = 20 each). Example of a DAB‐stained photomicrograph showing the expression of the GluN2B subunit in a horizontal section of the hippocampus. The regions of interest in the m.ml are marked by outlines. (A–C) Significant differences are marked with asterisks: **p < 0.01; ****p < 0.0001. GluN1, GluN2A, and GluN2B subunit expression in gcl, o.ml, and i.ml are shown in Supplementary Figure 4. Statistical results are summarized in Supplementary Table 2.

We then examined the relative content of GluN1, GluN2A, and GluN2B subunits of the NMDAR in the molecular and granule cell layer (Figure 5, Supplementary Figure 4, Supplementary Table 2). The middle molecular layer is the region where the MPP builds synapses with the DG (Groen, Kadish, and Wyss 2002). Examination of GluN1 revealed a higher expression in the middle molecular layer of the sDG compared to the iDG (unpaired *t* test: t_(36)_ = 3.450; *p* < 0.01, *R* ^2^ = 0.2484; *N* = 19 each; Figure 5A). A similar result was detected for GluN2A (Figure 5B; *N* = 20 each) and GluN2B subunits (Figure 5C; *N* = 20 each; unpaired *t* tests: GluN2A: t_(38)_ = 2.745; *p* < 0.01, *R* ^2^ = 0.1655; GluN2B: t_(38)_ = 5.962; *p* < 0.0001, *R* ^2^ = 0.4833). Examination of NMDAR subunit expression in the outer molecular layer revealed similar results to those for the middle molecular layer (Supplementary Figure 4, Supplementary Table 2). By contrast, no difference between iDG and sDG were detected in the granule cell layer for all subunits, whereas in the inner molecular layer, expression of GluN1 and GluN2B subunits was significantly different (Supplementary Figure 4, Supplementary Table 2). Overall, expression of all three subunits was higher in the middle and outer molecular layers of the sDG compared to the iDG.

## 4 Discussion

This study is the first to compare frequency‐dependent synaptic plasticity in MPP synapses of the iDG and sDG of the dorsal DG of freely behaving rats. We observed that the magnitude of LTD induced by LFS of the MPP at 1 Hz, as well as of LTP induced by HFS at 400 Hz, are weaker in the iDG compared to the sDG. HFS at 200 Hz induces no change in synaptic transmission in the iDG, whereas LTP in the sDG can be easily evoked by this frequency. Frequencies close to θm (e.g., 5 and 10 Hz) (Collitti‐Klausnitzer et al. 2021; Twarkowski, Hagena, and Manahan‐Vaughan 2016), that is, the putative modification threshold for a transition between frequency‐dependent LTD and LTP (Bienenstock, Cooper, and Munro 1982; Stanton 1996), when applied to the MPP, resulted in a transient depression followed by a late synaptic potentiation in both blades of the dorsal DG. Patch clamp recordings revealed a lower maximal firing frequency of granule cells of the iDG. Moreover, specific AP properties differed between blades. Immunohistochemical analysis of the expression of NMDAR subunits in the middle molecular layer of the DG where the MPP forms synapses (Steward 1976; Groen, Kadish, and Wyss 2002), revealed a lower amount of GluN1, GluN2A, and GluN2B subunits in the iDG compared to the sDG. These results strongly support that both blades of the dorsal DG exhibit unique properties, as well as different frequency‐dependencies and expression profiles of synaptic plasticity that may form the basis for their functional differentiation with regard to spatial and nonspatial information processing (Chawla et al. 2005; Erwin et al. 2020; Hoang, Aliane, and Manahan‐Vaughan 2018; Vazdarjanova et al. 2006).

Until now, synaptic plasticity in the iDG in the dorsal hippocampus has not been examined in detail in behaving rodents. Many papers do not state which blade was targeted for recordings, but implantation coordinates, or histological evidence provided about electrode locations, indicate that most studies on synaptic plasticity in the DG of behaving rodents focused on the dorsal sDG, whereas a few examined responses in the hilar region (Abraham et al. 1994; Altinbilek and Manahan‐Vaughan 2009; Blaise and Bronzino 2003; Frey and Frey 2009; Kenney and Manahan‐Vaughan 2013; Krug et al. 1983). In the present study, we specifically discriminated between the dorsal sDG and iDG and, as far as possible, conducted simultaneous recordings from both blades during MPP stimulation. We observed that the stimulus–response relationship was weaker in the sDG compared to the iDG in freely behaving rats. Differences in MPP inputs to the iDG and sDG are unlikely to explain these effects: axons from the EC simultaneously branch and innervate DG granule cells of both blades (Tamamaki and Nojyo 1993), the medial EC reportedly projects preferentially to the iDG rather than the sDG, whereas the lateral EC projects preferentially to the sDG (Tamamaki 1997; Wyss 1981). This suggests that granule cell excitability differs between blades, as reflected by the lower firing frequencies, higher AP threshold and the higher AHP minimum detected by means of patch clamp measurements of the iDG and sDG. One has to note, however, that chloride reversal currents, enabled by the patch pipette solution used in the present study (see Section 2) are likely to have altered the resting membrane potential via GABA‐A receptors, or other mechanisms involving chloride. Thus, the whole cell recordings in both DG blades were potentially influenced by such processes, albeit equivalently. No differences in firing frequency were detected in the stimulus range of 50–250 pA, as reported by others (Mishra and Narayanan 2020) and effects first emerged in the range of 300–700 pA. The current needed for cells to enter DB was lower in iDG. Local and global excitability properties of neurons will inevitably shape their synaptic plasticity responses. This is not only enabled by the generation of dendritic spikes, APs and their respective backpropagation by means of suprathreshold conductances (Golding, Staff, and Spruston 2002; Holthoff, Kovalchuk, and Konnerth 2006; Sjostrom and Hausser 2006), but is also mediated by subthreshold conductances that modulate synaptic potentials and support the temporal summation of these responses (Chen et al. 2006; Narayanan and Johnston 2010; Nolan et al. 2004). Structural differences between the iDG and sDG may also play a role in the determination of these properties: the sDG contains a higher density of dendritic spines than the iDG (Claiborne, Amaral, and Cowan 1990; Desmond and Levy 1985), specifically in the middle molecular layer that receives MPP input (Gallitano et al. 2016).

In line with the abovementioned findings and observations, we found that synaptic plasticity in the sDG induced by different afferent frequencies was comparable to previous studies, for example, HFS at 200 or 400 Hz applied to the MPP induces LTP in the sDG in behaving rats (Collitti‐Klausnitzer et al. 2021; Hagena and Manahan‐Vaughan 2024; Jeffery and Morris 1993; Manahan‐Vaughan, Braunewell, and Reymann 1998; Schulz et al. 1999). By contrast, the iDG does not exhibit significant changes in synaptic strength following 200 Hz stimulation, and although it expresses LTP in response to HFS at 400 Hz, the plasticity response is significantly smaller than LTP induced in sDG. In line with our findings, a study in anesthetized rats also reported the expression of LTP in the molecular layer of the iDG following 400 Hz stimulation of the angular bundle (Levy and Steward 1979). It is of course debatable whether MPP stimulation at 400 Hz reflects innate physiological processes, and therefore whether this finding is functionally meaningful. MPP afferent frequencies of 100 Hz generate weak LTP, or short‐term potentiation in vivo, but the coupling of weak afferent stimulation patterns with a spatial learning event generates LTP that lasts for at least 24 h (Hagena and Manahan‐Vaughan 2024). This suggests that the MPP is capable of transmitting high frequency impulses to the DG. Recordings from the lateral EC during trace conditioning of rats under anesthesia detected high frequency activity (> 100 Hz) that was coherent with hippocampal oscillations (de Oliveira, Dickson, and Reyes 2020). Ripple frequency volleys (140–200 Hz) also occur in the EC (Chrobak, Lorincz, and Buzsaki 2000). Furthermore, burst sequences in the range of 250–300 Hz have been detected in principal cells of the medial EC during exploratory behavior of mice (Csordas et al. 2020), suggesting that activity of this kind in the EC may generate the high frequencies described above that in turn drive the induction of persistent (> 24 h) LTP in the DG.

LTP that persists for longer than 24 h may subserve a role in the temporal linking of associative experiences in the generation of cognitive representations (Hagena and Manahan‐Vaughan 2024). Physiologically this may manifest itself in altered activity dynamics of specific neurons and neuronal networks. Correspondingly, the expression of LTP in conjunction with spatial learning, or the learning of an associative experience results in an increase of somatic immediate early gene expression in hippocampal neurons (Hoang, Aliane, and Manahan‐Vaughan 2018; Hoang, Boge, and Manahan‐Vaughan 2021), and the resultant stability of neuronal cFos expression in subpopulations of hippocampal CA neurons has led to the proposal that hippocampal engram neurons serve as the substrate for memory retention and retrieval (Jung et al. 2023; Liu et al. 2012; Mocle et al. 2024; Tayler et al. 2013). In line with this, memories in the DG are encoded in selected engram cells (Mishra and Narayanan 2022), supported by molecular mechanisms underlying intrinsic plasticity and synaptic plasticity (Brager and Johnston 2007; Fan et al. 2005; Frick, Magee, and Johnston 2004; Mishra and Narayanan 2022; Schmidt‐Hieber, Jonas, and Bischofberger 2004). Furthermore, it was recently reported that adult born granule cells exhibit a transient phase of lower thresholds for the induction of synaptic plasticity (Kennedy et al. 2024) that may serve to promote long‐term information storage. At least in the hours after LTP induction, structural plasticity that occurs at the level of dendritic spines may also support these processes (Bourne and Harris 2011).

Another form of persistent (> 24 h) synaptic plasticity comprises LTD (Hagena and Manahan‐Vaughan 2024). In the present study, we observed that LTD was evoked in both DG blades by LFS at 1 Hz, although the magnitude of responses was weaker in iDG compared to sDG synapses. This difference might be explained by the fact that spatially integrated EPSPs in the outer molecular layer tend to be smaller than in the middle molecular layer in the iDG compared to the sDG (Hayashi and Nonaka 2011), meaning that the propensity of the iDG to respond to 1 Hz MPP stimulation is less. At a functional level this may also be relevant for experience‐dependent information encoding (Chawla et al. 2005; Vazdarjanova et al. 2006), especially given the proposed role for hippocampal LTD in the processing and updating of novel spatial content information.

We also tested the effects of LFS at 5 or 10 Hz, given that these frequencies are in the range of θm that corresponds to the putative modification threshold for a transition between frequency‐dependent LTD and LTP (Bienenstock, Cooper, and Munro 1982; Stanton 1996; Twarkowski, Hagena, and Manahan‐Vaughan 2016). These frequencies are highly relevant for metaplasticity (Abraham and Bear 1996). They are typically too high to induce LTD and too low to induce LTP (Twarkowski, Hagena, and Manahan‐Vaughan 2016) but have an impact on a subsequent attempt to induce synaptic plasticity (Abraham and Bear 1996; Christie, Stellwagen, and Abraham 1995; Collitti‐Klausnitzer et al. 2021). They have been argued to lie within the “sliding threshold” for experience‐dependent induction of synaptic plasticity (Stanton 1996). Both frequencies resulted in transient depression followed by slow potentiation in both sDG and iDG, suggesting that physiological processes underlying metaplasticity in iDG and sDG may be similar.

As mentioned above, scrutiny using patch clamp recordings revealed that the maximal firing frequency of granule cells in the iDG is indeed smaller compared to the sDG. It seems likely that the smaller maximal firing frequencies that we detected using higher currents as well as the higher threshold needed to induce an AP and the smaller AP spike amplitude in the iDG may create constraints with regard to the induction of synaptic plasticity and/or limit its magnitude (Stanton and Sejnowski 1989; Wang, Rowan, and Anwyl 1997). This may be particularly relevant for the induction of LTD in the DG, especially considering that at least for the sDG, LTD does not require activation of NMDAR in adult behaving rats (Poschel and Manahan‐Vaughan 2007) or in juvenile rats in vitro (Wang, Rowan, and Anwyl 1997), although elevations of intracellular calcium levels are necessary for LTD induction in the DG (Wang, Rowan, and Anwyl 1997).

We observed that the iDG exhibits lower GluN subunit expression than the sDG. Poorer LTP responses in the iDG are likely to have been influenced by this weaker expression, given that in vitro studies have shown that activation of both GluN2A and GluN2B‐containing NMDAR support LTP induction in the DG (Vasuta et al. 2007) and mice lacking GluN2A show impaired LTP and LTD in DG (Kannangara et al. 2015). Furthermore, in sDG, LTP induced by 200 Hz HFS is NMDAR dependent in freely behaving rats, whereas LTP induced by 400 Hz stimulation recruits activation of both NMDAR and L‐type voltage‐gated calcium channels (VGCCs) (Manahan‐Vaughan, Braunewell, and Reymann 1998). Only 400 Hz HFS of the MPP resulted in LTP in iDG. Our immunohistochemical analyses did not differentiate the exact cellular location of GluN subunits, within or outside of functional synapses, thus a direct involvement of NMDARs in LTP in iDG still needs to be proven, even though it seems likely. One can only speculate as to whether L‐type VGCCs, or the activation of other receptors contributed to LTP in iDG. Although we did not assess this, others have reported that L‐type VGCCs are needed for neurogenesis in the DG (Marschallinger et al. 2015) and that the iDG exhibits higher neurogenesis than the sDG (Snyder, Ferrante, and Cameron 2012), hinting that these channels must be present in iDG and thus, could have contributed to LTP in this blade. T‐type calcium channels may also play a role in intrinsic excitability and the changing of LTP thresholds in adult born granule cells (Kennedy et al. 2024). In addition, AMPA receptor subunits reportedly differ in iDG and sDG, and expression may be modified by experience (Fraser, Makarowski, and McDonald 2021; Pandis et al. 2006). Experience‐dependent regulation of synaptic plasticity by metabotropic glutamate receptors of the iDG has also been reported, although effects were not compared with sDG (Rush et al. 2001). Inequity in the expression of peptide receptors have additionally been described (Sadamatsu et al. 1998). Distinct channels regulating intrinsic plasticity in the crest region of intermediate DG (Mishra and Narayanan 2022) may also differ in iDG and sDG. Assessing these aspects went beyond the scope of the present study but would provide valuable insights into the mechanisms underlying the functional differences we detected.

The difference in NMDAR subunit expression in both the middle and outer molecular layers between sDG and iDG suggest that not only the MPP‐iDG and MPP‐sDG pathways can be differentiated on the basis of subunit expression, AP properties and frequency dependent synaptic plasticity in vivo, but also that LPP‐iDG synapses and inputs are likely to be distinct from LPP‐sDG synapses (Hayashi and Nonaka 2011; Tamamaki 1997). In addition to the EC input, local circuitries enabled by feedforward, or feedback projections may also influence AP properties and cell excitability in the DG (Ewell and Jones 2010; Liu, Cheng, and Lien 2014). Thus, one could speculate that the iDG is subject to stronger inhibitory control than sDG, despite possibly receiving preferred input from the MEC (Tamamaki 1997), given that the maximal population spike and fEPSPs recorded in the present study were smaller compared to responses detected in sDG.

In conclusion, the results of this study strongly support the DG can be subdivided into at least two functionally distinct cellular and synaptic compartments that differ in their neuronal responsiveness, NMDAR expression levels, and their ability to express long‐term synaptic plasticity in the form of LTP and LTD. A clear result from this study is that long‐term synaptic plasticity, in the form of LTD and LTP, are distinct in sDG and iDG, both in terms of their frequency‐dependencies and their response profiles. The differences in the profiles and frequency dependency of persistent (> 24 h) forms of synaptic plasticity in sDG and iDG may reflect reported differences in information processing related to spatial and nonspatial experience. These findings also emphasize that the DG cannot be considered as a uniform entity when deciphering its role in information processing and storage.

## Author Contributions

The study was designed by D.M.‐V. and further developed with C.S. In vivo electrophysiological experiments and analysis: J.B. and C.S., patch clamp recordings and analysis: O.S., immunohistochemistry and analysis: V.D., D.M‐V., and C.S. interpreted the data and wrote the paper that was edited by all authors.

## Conflicts of Interest

The authors declare no conflicts of interest.

## Supporting information


**SUPPLEMENTARY FIGURE 1** Individual values of each animal for each time‐point recorded over the time course of the experiments in freely behaving rats. For each time‐point, evoked responses of each individual animal, as recorded from the supra‐ (sDG; light blue, circle) and infrapyramidal (iDG; dark blue, rhombus) blade of the dentate gyrus, are shown. (A) PS amplitude and fEPSP slope for sDG and iDG during test‐pulse stimulation of the medial perforant path (MPP). (B) PS amplitude and fEPSP slope for sDG and iDG before (−30 through 0 min) and after 1 Hz low‐frequency stimulation of MPP. (C) PS amplitude and fEPSP slope for both blades of the dentate gyrus before (−30 through 0 min) and after 5 Hz patterned afferent stimulation. (D) PS amplitude and fEPSP slope for sDG and iDG before (−30 through 0 min) and after 10 Hz patterned afferent stimulation of MPP. (E) PS amplitude and fEPSP slope for each animal recorded in sDG and iDG before (−30 through 0 min) and after 200 Hz high‐frequency stimulation (HFS) of the MPP. (F) PS amplitude and fEPSP slope for sDG and iDG before (−30 through 0 min) and after 400 Hz HFS. (A–F) Mean ± SEM for sDG and iDG are depicted as a frame of reference (Figures 2 and 3). The y‐axis is adjusted to optimize the presentation of the distribution for each experimental paradigm.

**SUPPLEMENTARY FIGURE 2** Cell and action potential properties recorded in granule cells of the supra‐ and infrapyramidal blade. (A–D) Passive and active membrane properties of granules cells do not differ between the supra‐ (sDG) and infrapyramidal (iDG) blades of the dentate gyrus. Individual data points represent patch clamp responses obtained from different neurons. (A) Input resistance, (B) resting membrane potential, (C) minimal current to induce an action potential, and (D) membrane time constant (tau) do not differ in granule cells of sDG (*n* = 19, *N* = 6) and iDG (*n* = 22, *N* = 6). (E–J) Several action potential properties are similar in granule cells of sDG (*N* = 19, *N* = 6) and iDG (*N* = 22, *N* = 6): (E) Peak amplitude and (F) depth of the afterhyperpolarization (AHP) from the threshold are not different between blades. (G) Total spike time, (H) width at half of the maximum amplitude, (I) time from the threshold to the peak, and (J) time from the peak of the action potential to the AHP are comparable in granule cells of sDG and iDG. (K) Example of the first action potential elicited in granule cells of sDG (pale blue) and iDG (dark blue). Calibration: Vertical bar: 20 mV, horizontal bar: 1 ms.

**SUPPLEMENTARY FIGURE 3** Firing frequency and spike frequency adaptation in granule cells of the supra‐ and infrapyramidal blade. (A) Firing frequency represented for each granule cell recorded in suprapyramidal (sDG, light blue, *n* = 19, *N* = 6) and infrapyramidal (iDG, dark blue, *n* = 22, *N* = 6) blade for each current step. Mean ± SEM for sDG and iDG are depicted for comparison with Figure 4D. (B) Examples of responses recorded for the 1 s current steps at 400, 500, 600 and 700 pA in a granule cell of the sDG and one of the iDG. Calibration: Vertical bar: 20 mV, horizontal bar: 100 ms. (C) The minimum current step that resulted in a depolarization block (DB) in some granule cells (sDG *n* = 7, iDG *n* = 10 cells) is significantly different between blades. No difference can be detected in (D) the plateau of DB and (E) resting membrane potential at the current step when DB was entered first.

**SUPPLEMENTARY FIGURE 4** Expression of GluN1, GluN2A, and GluN2B subunits in outer and inner molecular layer and granule cell layer of the supra‐ and infrapyramidal blade. (A) Examination of GluN1 subunits in the outer and inner molecular layer reveals a significantly lower expression in infrapyramidal (iDG) compared to the suprapyramidal (sDG) blade of the dentate gyrus (*N* = 19 each). In the granule cell layer the expression of the GluN1 subunit does not differ between blades. (B) GluN2A expression is significantly weaker in the outer molecular layer of the iDG compared to the sDG (*N* = 20 each), whereas the expression pattern in the inner molecular and granule cell layer does not differ. (C) In the outer and inner granule cell layer of iDG, GluN2B expression is significantly lower compared to sDG (*N* = 20 each). The expression in the granule cell layer does not differ. (A–C) Individual data points represent signal intensity measurements obtained from different hippocampal slices. Significant differences are marked with asterisks: * *p* < 0.05; *** *p* < 0.001, **** *p* < 0.0001. NMDAR subunit expression in the o.ml of sDG were published elsewhere (Collitti‐Klausnitzer et al. 2021).

**SUPPLEMENTARY TABLE 1** Outcome of statistical analysis of differences in responses to afferent stimulation of the infrapyramidal and suprapyramidal blades of the dentate gyrus.

**SUPPLEMENTARY TABLE 2** Summary of statistical analysis of GluN1, GluN2A, and GluN2B subunits.

## Data Availability

The data from this study are available from the corresponding author upon reasonable request.
