# Supplementary material for: The Suprapyramidal and Infrapyramidal Blades of the Dentate Gyrus Exhibit Different GluN Subunit Content and Dissimilar Frequency‐Dependent Synaptic Plasticity In Vivo
Source: Hippocampus. 2025 Feb 24;35(2):e70002. doi: 10.1002/hipo.70002 (PMC11850964; doi:10.1002/hipo.70002)
Supplement: Supplementary file 1 — SUPPLEMENTARY FIGURE 1 Individual values of each animal for each time‐point recorded over the time course of the experiments in freely behaving rats. For each time‐point, evoked responses of each individual animal, as recorded from the supra‐ (sDG; light blue, circle) and infrapyramidal (iDG; dark blue, rhombus) blade of the dentate gyrus, are shown. (A) PS amplitude and fEPSP slope for sDG and iDG during test‐pulse stimulation of the medial perforant path (MPP). (B) PS amplitude and fEPSP slope for sDG and iDG before (−30 through 0 min) and after 1 Hz low‐frequency stimulation of MPP. (C) PS amplitude and fEPSP slope for both blades of the dentate gyrus before (−30 through 0 min) and after 5 Hz patterned afferent stimulation. (D) PS amplitude and fEPSP slope for sDG and iDG before (−30 through 0 min) and after 10 Hz patterned afferent stimulation of MPP. (E) PS amplitude and fEPSP slope for each animal recorded in sDG and iDG before (−30 through 0 min) and after 200 Hz high‐frequency stimulation (HFS) of the MPP. (F) PS amplitude and fEPSP slope for sDG and iDG before (−30 through 0 min) and after 400 Hz HFS. (A–F) Mean ± SEM for sDG and iDG are depicted as a frame of reference (Figures 2 and 3). The y‐axis is adjusted to optimize the presentation of the distribution for each experimental paradigm. [file HIPO-35-0-s005.docx]

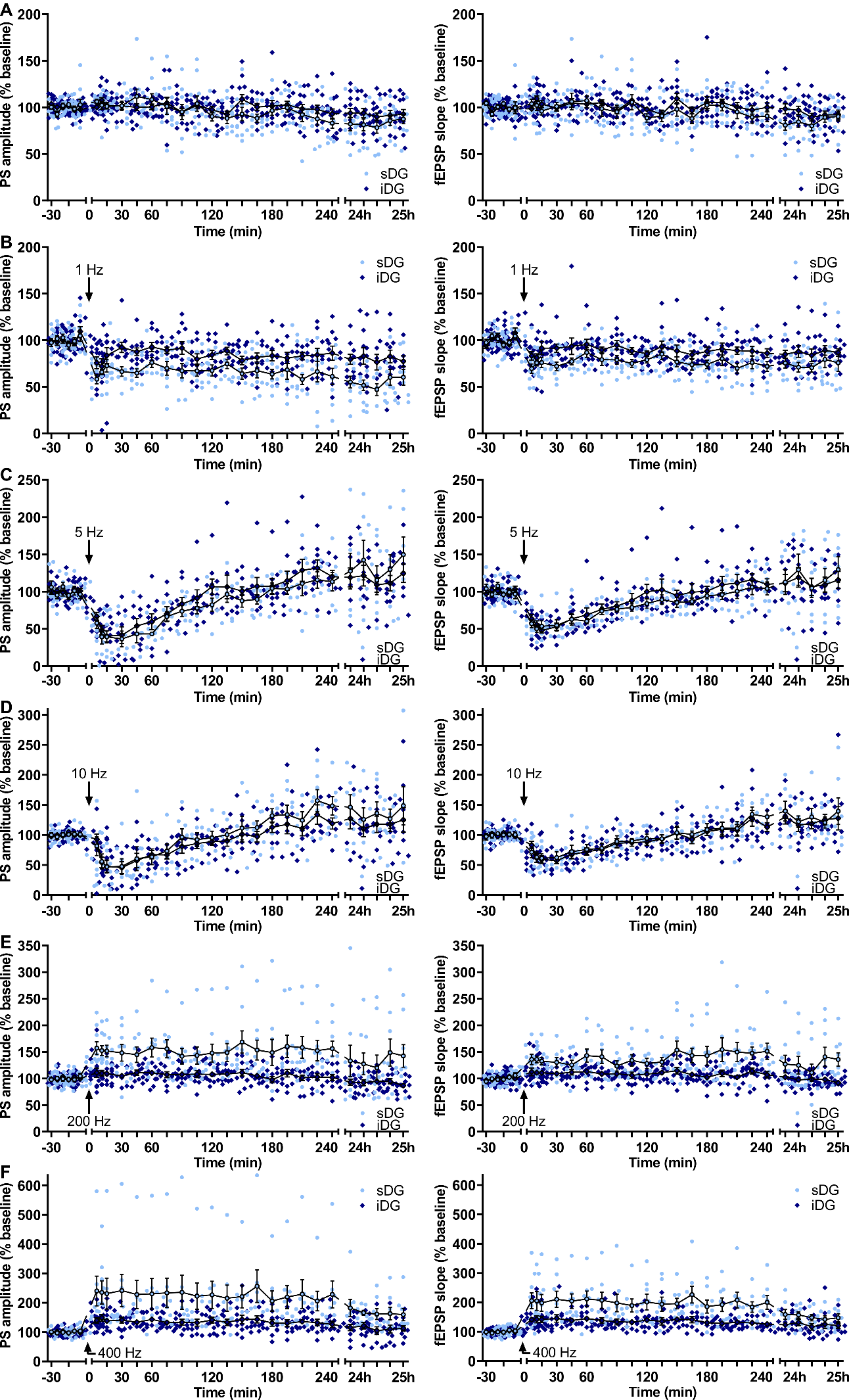


**Supplementary Figure 1**

**Individual values of each animal for each time-point recorded over the time course of the experiments in freely behaving rats.**

For each time-point, evoked responses of each individual animal, as recorded from the supra- (sDG; light blue, circle) and infrapyramidal (iDG; dark blue, rhombus) blade of the dentate gyrus, are shown.

A) PS amplitude and fEPSP slope for sDG and iDG during test-pulse stimulation of the medial perforant path (MPP).

B) PS amplitude and fEPSP slope for sDG and iDG before (-30 - 0 min) and after 1 Hz low-frequency stimulation of MPP.

C) PS amplitude and fEPSP slope for both blades of the dentate gyrus before (-30 - 0 min) and after 5 Hz patterned afferent stimulation.

D) PS amplitude and fEPSP slope for sDG and iDG before (-30 - 0 min) and after 10 Hz patterned afferent stimulation of MPP.

E) PS amplitude and fEPSP slope for each animal recorded in sDG and iDG before (-30 - 0 min) and after 200 Hz high-frequency stimulation (HFS) of the MPP.

F) PS amplitude and fEPSP slope for sDG and iDG before (-30 - 0 min) and after 400 Hz HFS.

A-F) Mean ± SEM for sDG and iDG are depicted as a frame of reference (Fig 2-3). The y-axis is adjusted to optimize the presentation of the distribution for each experimental paradigm.
