# Supplementary material for: The Suprapyramidal and Infrapyramidal Blades of the Dentate Gyrus Exhibit Different GluN Subunit Content and Dissimilar Frequency‐Dependent Synaptic Plasticity In Vivo
Source: Hippocampus. 2025 Feb 24;35(2):e70002. doi: 10.1002/hipo.70002 (PMC11850964; doi:10.1002/hipo.70002)
Supplement: Supplementary file 2 — SUPPLEMENTARY FIGURE 2 Cell and action potential properties recorded in granule cells of the supra‐ and infrapyramidal blade. (A–D) Passive and active membrane properties of granules cells do not differ between the supra‐ (sDG) and infrapyramidal (iDG) blades of the dentate gyrus. Individual data points represent patch clamp responses obtained from different neurons. (A) Input resistance, (B) resting membrane potential, (C) minimal current to induce an action potential, and (D) membrane time constant (tau) do not differ in granule cells of sDG (n = 19, N = 6) and iDG (n = 22, N = 6). (E–J) Several action potential properties are similar in granule cells of sDG (N = 19, N = 6) and iDG (N = 22, N = 6): (E) Peak amplitude and (F) depth of the afterhyperpolarization (AHP) from the threshold are not different between blades. (G) Total spike time, (H) width at half of the maximum amplitude, (I) time from the threshold to the peak, and (J) time from the peak of the action potential to the AHP are comparable in granule cells of sDG and iDG. (K) Example of the first action potential elicited in granule cells of sDG (pale blue) and iDG (dark blue). Calibration: Vertical bar: 20 mV, horizontal bar: 1 ms. [file HIPO-35-0-s002.docx]

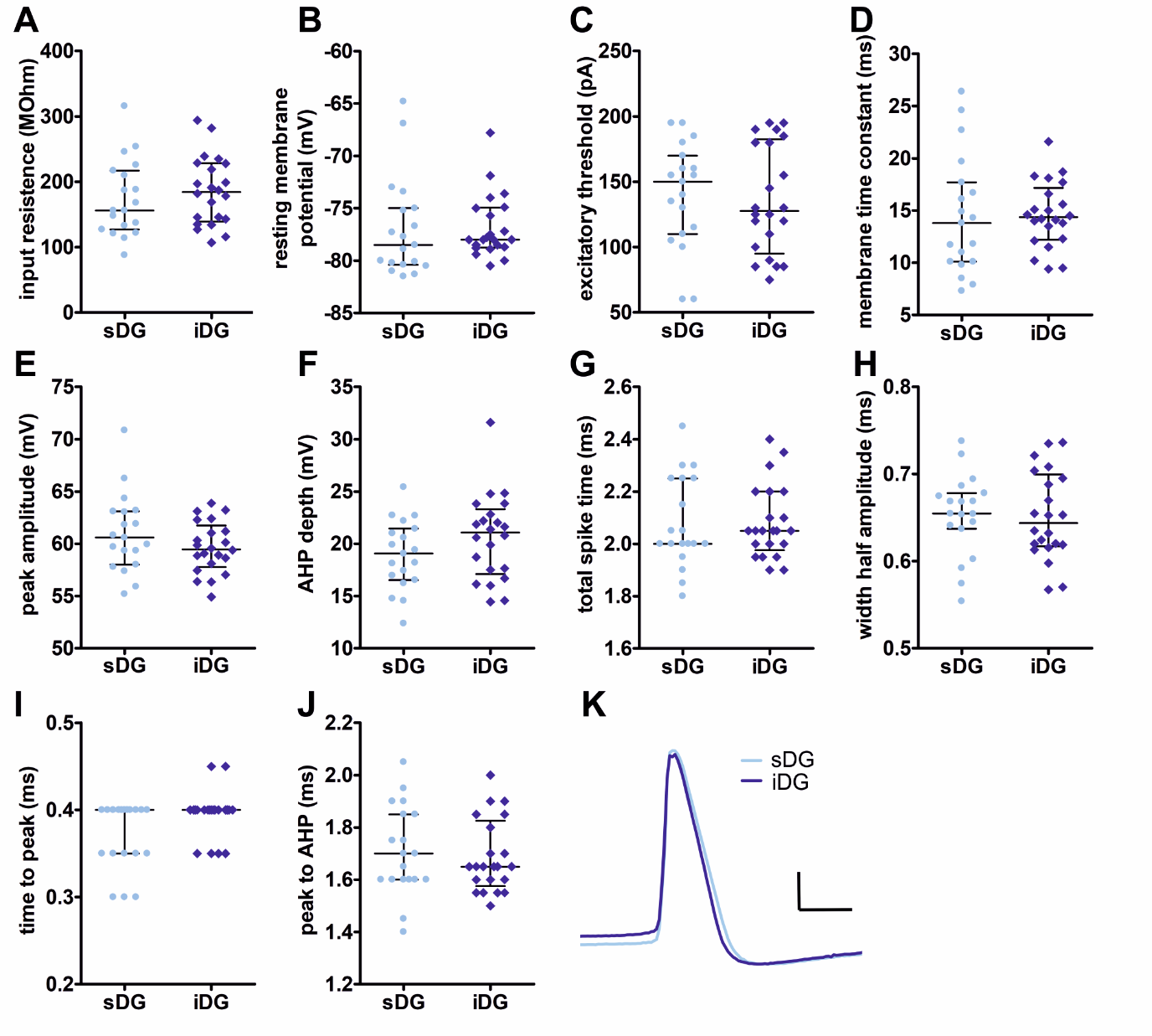


**Supplementary Figure 2**

**Cell and action potential properties recorded in granule cells of the supra- and infrapyramidal blade.**

A-D) Passive and active membrane properties of granules cells do not differ between the supra- (sDG) and infrapyramidal (iDG) blades of the dentate gyrus. Individual data points represent patch clamp responses obtained from different neurons.

A) Input resistance, B) resting membrane potential, C) minimal current to induce an action potential, and D) membrane time constant (tau) do not differ in granule cells of sDG (n = 19, N = 6) and iDG (n = 22, N = 6).

E-J) Several action potential properties are similar in granule cells of sDG (n = 19, N = 6) and iDG (n = 22, N = 6): E) Peak amplitude and F) depth of the afterhyperpolarization (AHP) from the threshold are not different between blades. G) Total spike time, H) width at half of the maximum amplitude, I) time from the threshold to the peak, and J) time from the peak of the action potential to the AHP are comparable in granule cells of sDG and iDG.

K) Example of the first action potential elicited in granule cells of sDG (pale blue) and iDG (dark blue). Calibration: Vertical bar: 20 mV, horizontal bar: 1 ms.
