# Supplementary material for: The Suprapyramidal and Infrapyramidal Blades of the Dentate Gyrus Exhibit Different GluN Subunit Content and Dissimilar Frequency‐Dependent Synaptic Plasticity In Vivo
Source: Hippocampus. 2025 Feb 24;35(2):e70002. doi: 10.1002/hipo.70002 (PMC11850964; doi:10.1002/hipo.70002)
Supplement: Supplementary file 3 — SUPPLEMENTARY FIGURE 3 Firing frequency and spike frequency adaptation in granule cells of the supra‐ and infrapyramidal blade. (A) Firing frequency represented for each granule cell recorded in suprapyramidal (sDG, light blue, n = 19, N = 6) and infrapyramidal (iDG, dark blue, n = 22, N = 6) blade for each current step. Mean ± SEM for sDG and iDG are depicted for comparison with Figure 4D. (B) Examples of responses recorded for the 1 s current steps at 400, 500, 600 and 700 pA in a granule cell of the sDG and one of the iDG. Calibration: Vertical bar: 20 mV, horizontal bar: 100 ms. (C) The minimum current step that resulted in a depolarization block (DB) in some granule cells (sDG n = 7, iDG n = 10 cells) is significantly different between blades. No difference can be detected in (D) the plateau of DB and (E) resting membrane potential at the current step when DB was entered first. [file HIPO-35-0-s006.docx]

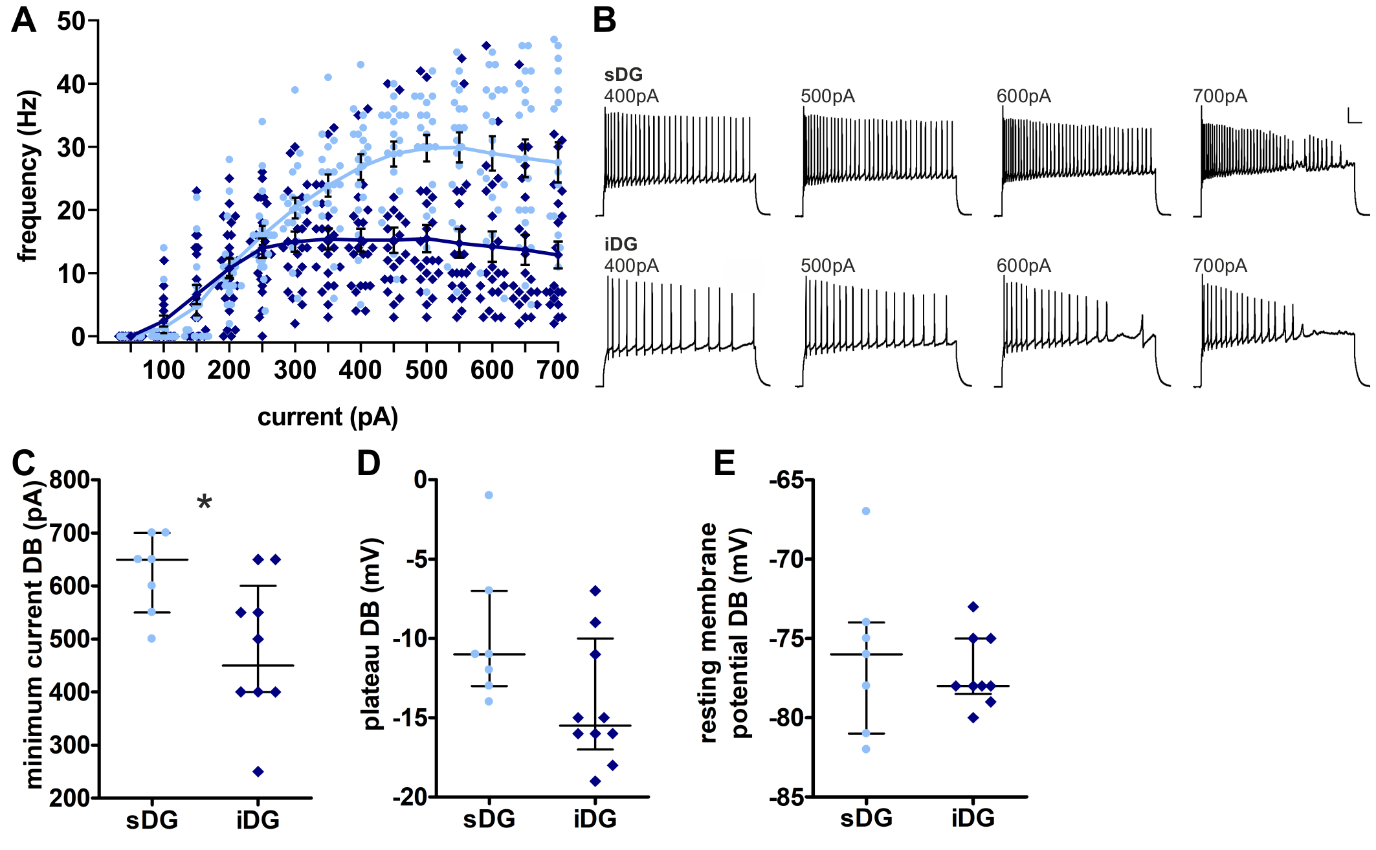


**Supplementary Figure 3**

**Firing frequency and spike frequency adaptation in granule cells of the supra- and infrapyramidal blade.**

A) Firing frequency represented for each granule cell recorded in suprapyramidal (sDG, light blue, n = 19, N = 6) and infrapyramidal (iDG, dark blue, n = 22, N = 6) blade for each current step. Mean ± SEM for sDG and iDG are depicted for comparison with Fig. 4D.

B) Examples of responses recorded for the 1 s current steps at 400, 500, 600 and 700 pA in a granule cell of the sDG and one of the iDG. Calibration: Vertical bar: 20 mV, horizontal bar: 100 ms.

C) The minimum current step that resulted in a depolarization block (DB) in some granule cells (sDG n = 7, iDG n = 10 cells) is significantly different between blades. No difference can be detected in D) the plateau of DB and E) resting membrane potential at the current step when DB was entered first.
