# Supplementary material for: The Suprapyramidal and Infrapyramidal Blades of the Dentate Gyrus Exhibit Different GluN Subunit Content and Dissimilar Frequency‐Dependent Synaptic Plasticity In Vivo
Source: Hippocampus. 2025 Feb 24;35(2):e70002. doi: 10.1002/hipo.70002 (PMC11850964; doi:10.1002/hipo.70002)
Supplement: Supplementary file 4 — SUPPLEMENTARY FIGURE 4 Expression of GluN1, GluN2A, and GluN2B subunits in outer and inner molecular layer and granule cell layer of the supra‐ and infrapyramidal blade. (A) Examination of GluN1 subunits in the outer and inner molecular layer reveals a significantly lower expression in infrapyramidal (iDG) compared to the suprapyramidal (sDG) blade of the dentate gyrus (N = 19 each). In the granule cell layer the expression of the GluN1 subunit does not differ between blades. (B) GluN2A expression is significantly weaker in the outer molecular layer of the iDG compared to the sDG (N = 20 each), whereas the expression pattern in the inner molecular and granule cell layer does not differ. (C) In the outer and inner granule cell layer of iDG, GluN2B expression is significantly lower compared to sDG (N = 20 each). The expression in the granule cell layer does not differ. (A–C) Individual data points represent signal intensity measurements obtained from different hippocampal slices. Significant differences are marked with asterisks: * p < 0.05; *** p < 0.001, **** p < 0.0001. NMDAR subunit expression in the o.ml of sDG were published elsewhere (Collitti‐Klausnitzer et al. 2021). [file HIPO-35-0-s004.docx]

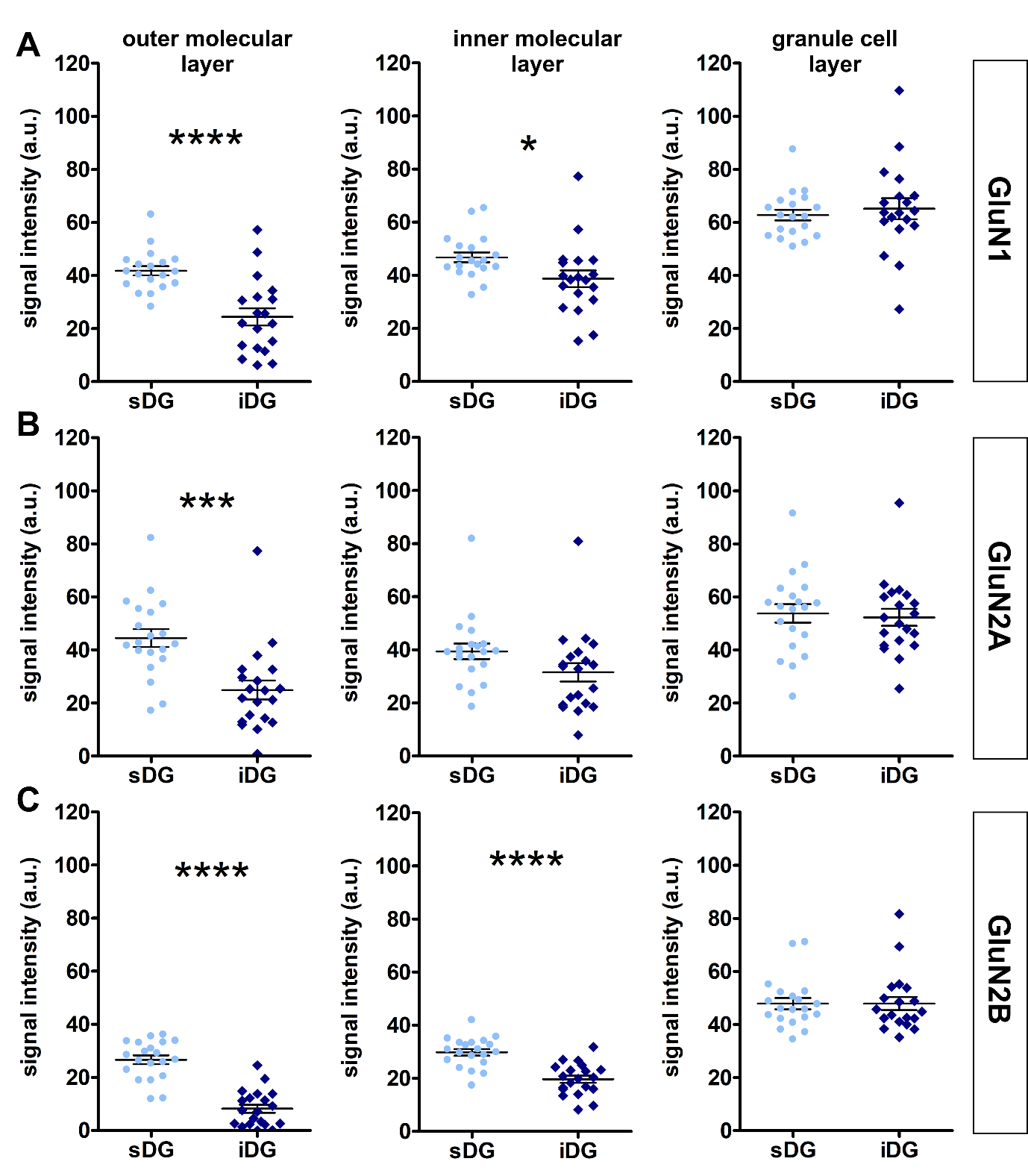


**Supplementary Figure 4**

**Expression of GluN1, GluN2A and GluN2B subunits in outer and inner molecular layer and granule cell layer of the supra- and infrapyramidal blade.**

A) Examination of GluN1 subunits in the outer and inner molecular layer reveals a significantly lower expression in infrapyramidal (iDG) compared to the suprapyramidal (sDG) blade of the dentate gyrus (N = 19 each). In the granule cell layer the expression of the GluN1 subunit does not differ between blades.

B) GluN2A expression is significantly weaker in the outer molecular layer of the iDG compared to the sDG (N = 20 each), whereas the expression pattern in the inner molecular and granule cell layer does not differ.

C) In the outer and inner granule cell layer of iDG, GluN2B expression is significantly lower compared to sDG (N = 20 each). The expression in the granule cell layer does not differ.

A-C) Individual data points represent signal intensity measurements obtained from different hippocampal slices. Significant differences are marked with asterisks: * p < 0.05; *** p < 0.001, **** p < 0.0001. The results of the o.ml of sDG were published elsewhere (Collitti-Klausnitzer et al., 2021).
