# Supplementary material for: The Suprapyramidal and Infrapyramidal Blades of the Dentate Gyrus Exhibit Different GluN Subunit Content and Dissimilar Frequency‐Dependent Synaptic Plasticity In Vivo
Source: Hippocampus. 2025 Feb 24;35(2):e70002. doi: 10.1002/hipo.70002 (PMC11850964; doi:10.1002/hipo.70002)
Supplement: Supplementary file 5 — SUPPLEMENTARY TABLE 1 Outcome of statistical analysis of differences in responses to afferent stimulation of the infrapyramidal and suprapyramidal blades of the dentate gyrus. [file HIPO-35-0-s001.docx]

**Supplementary Table 1**

**Outcome of statistical analysis of differences in responses to afferent stimulation of the infrapyramidal and suprapyramidal blades of the dentate gyrus.**

Interaction effect of time*groups for PS amplitude and fEPSP slope for test-pulse stimulation and patterned afferent stimulation in suprapyramidal (sDG) and infrapyramidal (iDG) blade of the dentate gyrus.

| **Time*group** | **PS amplitude** | | **fEPSP slope** | |
| --- | --- | --- | --- | --- |
|  | **F** | **p** | **F** | **p** |
| Test-pulse (sDG vs iDG) | F_(22,462)_ = 1.110 | 0.331 | F_(22,462)_ = 0.698 | 0.843 |
| 1Hz (sDG vs iDG) | F_(22,396)_ = 0.595 | 0.927 | F_(22,396)_ = 0.337 | 0.998 |
| 1Hz (test-pulse vs sDG) | F_(22,374)_ = 0.867 | 0.64 | F_(22,374)_ = 1.176 | 0.265 |
| 1Hz (test-pulse vs iDG) | F_(22,484)_ = 1.524 | 0.061 | F_(22,484)_ = 1.322 | 0.150 |
| 5Hz (sDG vs iDG) | F_(22,308)_ = 0.827 | 0.691 | F_(22,308)_ = 0.518 | 0.966 |
| 5Hz (test-pulse vs sDG) | F_(22,330)_ = 12.051 | **< 0.0001** | F_(22,330)_ = 8.640 | **< 0.0001** |
| 5Hz (test-pulse vs iDG) | F_(22,440)_ = 16.388 | **< 0.0001** | F_(22,440)_ = 13.169 | **< 0.0001** |
| 10Hz (sDG vs iDG) | F_(22,308)_ = 0.497 | 0.973 | F_(22,308)_ = 0.278 | 1.000 |
| 10Hz (test-pulse vs sDG) | F_(22, 330)_ = 12.924 | **< 0.0001** | F_(22,330)_ = 9.688 | **< 0.0001** |
| 10Hz (test-pulse vs iDG) | F_(22,440)_ = 14.012 | **< 0.0001** | F_(22,440)_ = 14.225 | **< 0.0001** |
| 200Hz (sDG vs iDG) | F_(22,418)_ = 0.637 | 0.897 | F_(22,418)_ = 1.182 | 0.259 |
| 200Hz (test-pulse vs sDG) | F_(22,374)_ = 0.718 | 0.822 | F_(22,374)_ = 0.933 | 0.551 |
| 200Hz (test-pulse vs iDG) | F_(22,506)_ = 0.923 | 0.564 | F_(22,506)_ = 1.106 | 0.336 |
| 400Hz (sDG vs iDG) | F_(22,374)_ = 1.251 | 0.201 | F_(22,374)_ = 1.673 | **< 0.05** |
| 400Hz (test-pulse vs sDG) | F_(22,352)_ = 2.073 | **< 0.01** | F_(22,352)_ = 2.115 | **< 0.01** |
| 400Hz (test-pulse vs iDG) | F_(22,484)_ = 1.065 | 0.382 | F_(22,484)_ = 0.951 | 0.527 |

*Level of significance: p < 0.05; p < 0.01, and p < 0.0001*
