# Supplementary material for: The Suprapyramidal and Infrapyramidal Blades of the Dentate Gyrus Exhibit Different GluN Subunit Content and Dissimilar Frequency‐Dependent Synaptic Plasticity In Vivo
Source: Hippocampus. 2025 Feb 24;35(2):e70002. doi: 10.1002/hipo.70002 (PMC11850964; doi:10.1002/hipo.70002)
Supplement: Supplementary file 6 — SUPPLEMENTARY TABLE 2 Summary of statistical analysis of GluN1, GluN2A, and GluN2B subunits. [file HIPO-35-0-s003.docx]

**Supplementary Table 2**

**Summary of statistical analysis of GluN1, GluN2A and GluN2B subunits.**

An unpaired t-test was performed to examine expression differences in GluN1 (N = 19), GluN2A (N = 20) and GluN2B (N = 20) subunits of the NMDA receptor of the infra- (iDG) compared to the suprapyramidal (sDG) blade.

|  | **t** | **p-value** | **R² (eta^2^)** | **Difference between means ± SEM** | **95% CI** |
| --- | --- | --- | --- | --- | --- |
| **GluN1** |  |  |  |  |  |
| o. ml | 4.695 | **< 0.0001** | 0.3798 | -17.38 ± 3.701 | -24.89 to -9.873 |
| m. ml | 3.450 | **< 0.01** | 0.2484 | -12.79 ± 3.708 | -20.31 to -5.272 |
| i. ml | 2.188 | **< 0.05** | 0.1174 | -8.051 ± 3.679 | -15.51 to -0.5892 |
| gcl | 0.5480 | 0.5871 | 0.008274 | 2.423 ± 4.422 | -6.544 to 11.39 |
|  |  |  |  |  |  |
| **GluN2A** |  |  |  |  |  |
| o. ml | 3.983 | **< 0.001** | 0.2945 | -19.56 ± 4.910 | -29.49 to -9.616 |
| m. ml | 2.745 | **< 0.01** | 0.1655 | -13.57 ± 4.943 | -23.57 to -3.562 |
| i. ml | 1.738 | 0.0902 | 0.07367 | -7.898 ± 4.543 | -17.10 to 1.299 |
| gcl | 0.3123 | 0.7565 | 0.002560 | -1.470 ± 4.706 | -11.00 to 8.057 |
|  |  |  |  |  |  |
| **GluN2B** |  |  |  |  |  |
| o. ml | 8.281 | **< 0.0001** | 0.6435 | -18.43 ± 2.225 | -22.93 to -13.92 |
| m. ml | 5.962 | **< 0.0001** | 0.4833 | -12.63 ± 2.118 | -16.91 to -8.340 |
| i. ml | 5.503 | **< 0.0001** | 0.4435 | -10.12 ± 1.839 | -13.84 to -6.398 |
| gcl | 0.01063 | 0.9916 | <0.0001 | 0.03468 ± 3.261 | -6.567 to 6.636 |

*Difference between means ± SEM: calculated as iDG - sDG*

*95% CI: 95% confidence interval of difference between means*

*o. ml: outer molecular layer, m. ml: middle molecular layer, i. ml: inner molecular layer, gcl: granule cell layer*

*Level of significance: p < 0.05; p < 0.01, p < 0.001, and p < 0.0001*
